# Conjugative Transfer of Disease‐Encoding Plasmid Variants in *Serratia* spp. Alter Production of Enzymes and Virulence Properties

**DOI:** 10.1111/1758-2229.70292

**Published:** 2026-02-10

**Authors:** Amy L. Vaughan, Travis R. Glare, Charles A. Hefer, Mark R. H. Hurst

**Affiliations:** ^1^ Bio‐Protection Research Centre, Lincoln University Christchurch New Zealand; ^2^ AgResearch Bioeconomy Science Institute, Resilient Agriculture, Lincoln Research Centre Christchurch New Zealand; ^3^ Lincoln Agritech Ltd, Lincoln University Christchurch New Zealand; ^4^ AgResearch Bioeconomy Science Institute, Bioinformatics, Analytics and Modelling, Lincoln Research Centre Christchurch New Zealand

## Abstract

Some strains of *Serratia entomophila, S. proteamaculans* and *S. quinivorans* (Enterobacterales: Yersiniaceae) are entomopathogens of the New Zealand pasture pest *Costelytra giveni* (Coleoptera: Scarabaeidae). Virulence is encoded by variants of the amber disease‐associated plasmid (pADAP), collectively termed *Serratia* transmissible adaptive megaplasmids (STAMPs), whose diverse insect‐active complexes impart hypervirulence to chronic pathotypes. An estimated 40%–60% of New Zealand *Serratia* are plasmid‐free non‐virulent conspecifics to STAMP‐carrying entomopathogens, implying a complex evolutionary relationship between the plasmid, host and disease. To further define this relationship, plasmids from chronic and hypervirulent pathotypes were conjugated into recipient strains, allowing experimental comparison of virulence relative to donor and naïve strains. Through competitive bioassays and plate‐based enzyme assays, transconjugants (strains selectively conjugated with donor plasmids) showed altered enzymatic activity and variable disease phenotypes. Transconjugants were also found to have reduced fitness, outcompeted by naïve plasmid‐free and native plasmid‐bearing strains within the host and in vitro cultures, suggesting a degree of coevolution. Transcriptomic analysis comparing naïve strains and transconjugants revealed differentially expressed genes associated with virulence, including plasmid‐encoded anti‐feeding prophage (Afp) genes and chromosomal chitinases and proteases. Results further support that STAMPs have speciated to their host chromosome and that naturally occurring *Serratia* plasmid‐containing isolates have coevolved accordingly.

## Graphical abstract

Analysis of plasmid transconjugants relative to plasmid‐free and plasmid‐carrying *Serratia* spp. demonstrates erratic disease phenotypes and reduced fitness. Transconjugants also show reduced ability to degrade substrates associated with virulence, supported by transcriptomic data. These results further support that *Serratia* plasmids have speciated to their host chromosome and have coevolved.

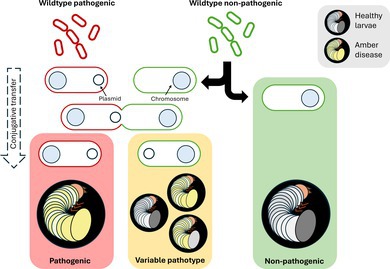

## 1 Introduction

As members of the Enterobacteriaceae, *Serratia* are ubiquitous, free‐living Gram‐negative proteobacteria within a broad genus. They comprise diverse facultative pathogens of both human and animal hosts. *Serratia entomophila* , *S. proteamaculans* and *S. quinivorans* (Enterobacterales: Yersiniaceae) are known entomopathogens of larvae of the New Zealand grass grub *Costelytra giveni* (Coleoptera: Scarabaeidae) and manuka beetle *Pyronota* spp. (Coleoptera: Scarabaeidae) (Hurst et al. 2018, 2023; Jackson et al. 1993). The *C. giveni* specific *S. entomophila* type strain *Se*A1MO2 has been the subject of extensive research, causing a chronic infection within challenged larvae that then take several weeks to die (Jackson et al. 1993). Subjecting *Se*A1MO2 to heat eliminated a large plasmid to form the plasmid‐free strain avirulent strain *Se*5.6. Based on this, the *Se*A1MO2, 153 Kb plasmid was identified as crucial for amber disease in larvae and subsequently designated pADAP for amber disease‐associated plasmid (Glare et al. 1993). The pADAP plasmid encodes two virulence‐associated regions, (i) the *S. entomophila* pathogenicity Sep locus, that encodes an insect active toxin complex (Tc) responsible for gut clearance and amber colouration in larvae (Hurst et al. 2000) and (ii) a phage tail‐like structure termed the anti‐feeding prophage Afp that, post ingestion, leads to cessation of feeding by the challenged larvae (Hurst et al. 2004).

Several *S. proteamaculans* and *S. quinivorans* Tc encoding clusters have since been described (Hurst et al. 2011; Dodd et al. 2006), some of which are active against both grass grub and manuka beetle larvae (Hurst et al. 2021). The most virulent *Serratia* is the *S. proteamaculans* strain *Sp*AGR96X that encodes an Afp variant termed AfpX. Grass grub and manuka beetle larvae challenged with *Sp*AGR96X die within 5–12 days after ingestion (Hurst et al. 2018). The diversity of these Tc and Afp virulence clusters and their differing pathotypes suggests a degree of speciation of these pathogens with their scarab hosts.

Within the species *S. entomophila*, the pADAP plasmids share > 99% nucleotide identity across their full length, whereas in contrast, the *S. proteamaculans* and *S. quinivorans* pADAP plasmids are highly heterogeneous, despite sharing a common ~63 kb plasmid backbone (Sitter et al. 2021). Based on the presence of a conserved backbone, these pADAP variants which are found across these three *Serratia* species are termed STAMPs for *Serratia* transmissible adaptive mega‐plasmids (Sitter et al. 2021). While STAMPs impart the disease phenotype, between 40% and 60% of *Serratia* strains in the soil are non‐pathogenic and are likely plasmid‐free (Jackson et al. 2001). Examples of naturally occurring plasmid‐free isolates include *S. proteamaculans* strain *Sp*3041 and *S. entomophila Se*477. It has been proposed that due to their reduced metabolic load, plasmid‐free *Serratia* strains may outcompete their pathogenic conspecifics (Godfray et al. 1999).

At the chromosomal level genomic comparisons based on average nucleotide identity (ANI), Williams et al. (2022) validated that the phylogroups of *S. proteamaculans, S. quinivorans* and *S. entomophila* comprised three distinct lineages. The *S. entomophila* strain *Se*626 was explored by Vaughan et al. (2022), where few signatures of horizontal gene transfer were identified within the chromosome, indicative of potential speciation at the time pADAP was acquired. Further work examining the lineage of *S. entomophila* defined the pan‐genome as ‘closed’ with no noted acquisitions of foreign DNA, limiting genetic diversity (Williams et al. 2022). In contrast, the diverse phenotype of disease found caused by *S. proteamaculans* and *S. quinivorans*, including the hypervirulent *S* *p*AGR96X (Sitter et al. 2021; Hurst et al. 2018), suggests the genome is largely heterogeneous, potentially contributing to its more variable pathovar (Williams et al. 2022).

The ability of *S. entomophila* and *S. proteamaculans* to acquire virulence by conjugation of pADAP family plasmids has been previously demonstrated by Grkovic et al. (1995) and Sitter et al. (2021). The latter study also showed that STAMPs remain highly stable following transfer into non‐native, recipient strains, with 100% plasmid retention over a 10‐day period. Based on genomic analysis, Sitter et al. (2021) proposed that these *Serratia* conjugative plasmids evolved from an ancestral plasmid with adaptive qualities to suit the ecology of the bacterial host. Such compensatory adaptations could mitigate fitness costs of plasmid carriage, such as mutations to select genes involved in regulatory systems (Harrison et al. 2015; San Millan et al. 2015). Comparative chromosomal analysis of *S. proteamaculans, S. quinivorans* and *S. entomophila* showed no plasmid‐associated chromosomal adaptations, or accessory genes such as chitinases, lipases and proteases that may impart a yet to be determined benefit were linked to enhanced pathogenicity or bacterial fitness (Vaughan 2021). This indicates that virulence is primarily plasmid‐encoded, and that plasmid stability is maintained independently of chromosomal modification (Vaughan 2021).

To investigate the evolutionary trajectory of STAMPs and their contribution to virulence, representative chronic and hypervirulent plasmids were transferred into plasmid‐free and heat‐cured variants of *Serratia* strains. The resulting transconjugants (a bacterium that has received genetic material by conjugation with another bacterium) were evaluated for virulence‐associated traits through various assays including those assessing for chitinase, lipase and siderophore production as well as for potential plant association ability via the screening for indole‐3‐acetic acid (IAA) production. To define any competition between naïve isolates and their transconjugant derivatives, a range of competition assays, plasmid stability tests, transcriptomic analyses and LC_50_/LT_50_ bioassays were used. The resultant findings further help to elucidate the evolutionary pressures on *Serratia* and identify any markers suggestive of host‐specific and/or niche adaptation.

## 2 Experimental Procedures

### 2.1 Cultures

Unless otherwise stated, cultures were grown in 3 mL of Luria‐Bertani (LB) broth for 16 h at 37°C for *Escherichia coli*, and 30°C for isolates of *S. entomophila*, *S. proteamaculans* and *S. quinivorans* and their derivatives (Table 1). Cultures were incubated with shaking at 250 rpm in a Ratek orbital incubator. Antibiotic concentrations used for *Serratia* were spectinomycin 100 μg mL^−1^, tetracycline 30 μg mL^−1^, kanamycin 100 μg mL^−1^, ampicillin 100 μg mL^−1^, chloramphenicol 90 μg mL^−1^ and for *E. coli* were ampicillin 100 μg mL^−1^, kanamycin 50 μg mL^−1^ and tetracycline 30 μg mL^−1^. For the growth of *E. coli* strain ST18, 50 μg/mL^−1^ of 5‐aminolevulinic acid was added. LB and M9 (glucose) minimal growth media were prepared as described in Elbing and Brent (2019). To validate the presence of *S. entomophila*, selective caprylate‐thallous agar (CTA), DNase and itaconate (ITA) plates were prepared and applied as outlined by O'Callaghan and Jackson (1993).

**TABLE 1:** Strains and transconjugants used in this study.

Naïve and heat‐cured (plasmid‐free) strains used as donors and recipient				
Species	Strain	Virulence	Plasmid	
Serratia entomophila	*Se*iDIA	Chronic	piDIA	
	*Se*iDIA^−^	Non‐pathogenic	Heat‐cured *Se*iDIA (plasmid‐free)	
	*Se*477*	Non‐pathogenic	Plasmid‐free	
	*Se*A1M02*	Chronic	pADAP	
	*Se*626	Chronic	p626	
	*Se*5.6	Non‐pathogenic	Heat‐cured *Se*A1M02 (plasmid‐free)	
*Serratia proteamaculans*	*Sp*3041	Non‐pathogenic	Plasmid‐free	
	*Sp*AGR96X	Hypervirulent	pAGR96X	

Donor plasmids/strains and the corresponding transconjugant or heat‐cured derivatives constructed through this study. Antibiotic marker cassettes for donor plasmid and recipient strain displayed in brackets as Spec, Tet, Kan or Cm.				
Donor plasmid	Donor strain	Recipient strain	Resulting transconjugant	Heat cured
p626 (Cm)	*Se*626	*Se*iDIA (Tet)	*Se*iDIA (p626)	—
pAGR96X (Cm)	*Sp*AGR96X	*Se*iDIA (Tet)	*Se*iDIA (pAGR96X)	—
—	—	*Se*iDIA (Tet)	*Se*iDIA^−^	Plasmid‐free control
p626 (Cm)	*Se*626	*Se*477 (Tet)	*Se*477 (p626)	—
pAGR96X (Cm)	*Sp*AGR96X	*Se*477(Tet)	*Se*477 (pAGR96X)*	—
pADAP (Kan)	*Se*A1M02	*Se*477 (Tet)	*Se*477 (pADAP)*	—
pAGR96X (Cm)	*Sp*AGR96X	*Se*5.6 (Spec)	*Se*5.6 (pAGR96X)	—
p626 (Cm)	*Se*626	*Se*5.6 (Spec)	*Se*5.6 (p626)	—
pADAP (Kan)	*Se*A1M02	*Se*5.6 (Spec)	*Se*5.6 (pADAP)	—
pAGR96X (Cm)	*Sp*AGR96X	*Sp*3041 (Spec)	*Sp*3041 (pAGR96X)	—

* Denotes isolates and derivatives further utilised for transcriptomics.

For microbial growth curves, 3 mL LB broth cultures were initially grown for approximately 16 h (~1 × 10^9^ CFU). Starting concentrations were then equalised through the addition of LB broth to a CFU of ~1 × 10^7^ CFU/mL, and subsequent CFUs were determined by serial dilution. An aliquot of 500 μL of the equilibrated culture was then pelleted (8000*g* for 3 min 4°C) and the pellet resuspended in 500 μL phosphate‐buffered saline (PBS) before being independently inoculated into three flasks containing either 50 mL of LB broth or three flasks containing M9 (glucose) per isolate. CFUs were determined using serial dilutions prepared in PBS buffer by taking 1 mL samples at the time of inoculation and 1, 2, 4, 8, 16 and 24 h post‐inoculation (hpi). OD_600_ was measured in triplicate per biological replicate (two per sample) at each of these time points using a Bio‐Rad SmartSpec Plus Spectrophotometer.

### 2.2 Isolates Used and Constructed Through the Study

Isolates used in this study were selected based on their virulence level (hypervirulent, chronic, non‐pathogenic), species representation ( *Serratia entomophila* : *Se, Serratia proteamaculans: Sp*) (Table 1) and nucleotide similarity (> 99.5% for *S. entomophila* strains). The plasmid‐cured derivatives of isolates *Se*iDIA (termed *Se*iDIA^−^), *Se*A1M02 (*Se*5.6; Glare et al. 1993) and the naturally occurring plasmid‐free isolates *Se*477 and *Sp*3041 were used in the study as recipient strains for donor plasmids (Table 1). Bacterial conjugation was undertaken on LB agar as previously described (Sitter et al. 2021), resulting in transconjugant strains derivatives that have received tagged, donated plasmid material from a donor strain. Transconjugants are designated by the recipient strain name followed by the plasmid in parentheses (e.g., *Se*iDIA (pAGR96X) refers to the *Se*iDIA recipient carrying the donor plasmid pAGR96X) (Table 1). The transconjugant *Se*5.6 (pADAP) served as a control and a model comparison for the relationship between the plasmid and its host chromosome, as well as to enable the identification of any unknown implications caused by the removal of the virulence plasmid through heat curing, due to it being a restoration of the original pADAP variant to the host *Se*5.6 chromosome (Table 1). Multiple attempts to derive a plasmid‐cured isolate of *Sp*AGR96X were unsuccessful and attempts to conjugate the *Se* plasmids (pADAP and p626) to *Sp*3041 were also unsuccessful.

Plasmid and chromosome tags were constructed as described by Watson (2022). Conjugation was undertaken using the *E. coli* donor strain ST18, following the method of Martínez‐García and de Lorenzo (2011). Transconjugants were plated on antibiotic selective media for the recombined antibiotic cassette. Colonies were then patched to counter‐select for the suicide plasmid. Prospective transconjugants were then PCR validated using internal facing primers positioned external to the recombined region (Table 2). Prospective transconjugants were selected for using antibiotic markers located on plasmid and the chromosome (Watson 2022), from where the plasmid was visualised using the method of Kado and Liu (1981) and the strain profile determined using BOXA1R PCR (Versalovic 1994).

**TABLE 2:** Primers and templates used in this study.

Name	Sequence (5′–3′)	Description	Template
AfpX_f	GGGCAGTATTCCGCTGCAGACGG	Validation of pAGR96X antibiotic (Cm) insert	pAGR96X
AfpX_r	TCTAGCCATCATGGTGCGGCAACC	Validation of pAGR96X antibiotic (Cm) insert	pAGR96X
repA_f	TGGAGGGGAACGATCTTCTTGAGG	*repA*	pADAP
repA_r	GCCCCACTTTCTTGTACCATCCAG	*repA*	
M13_F	GTAAAACGACGGCCAGT	Sequencing	pGEM
M13_R	GCGGATAACAATTTCACACAGG	Sequencing	pGEM
M13_pUC_r	GTTTTCCCAGTCACGAC	Sequencing	pUC19
M13_pUC_r	CAGGAAACAGCTATGAC	Sequencing	pUC19
pHP45Omega_Spec_amp_F	AAACCCTCACTGATCCGCATG	Spec cassette	pHP45
pHP45Omega_Spec_amp_R	AGACTTGACCTGATAGTTTGGCTG	Spec cassette	pHP45
BacF	AGGTGACGGTGGTAATGG	Region A	
BacR	TGGAAGTGCATATCCATA	Region A	
BOX A1R	CTACGGCAAGGCGACGCTGACG		

### 2.3 Plate Assays

Unless otherwise stated, bacterial colonies in plate assays were patched in triplicate on three independent plates and incubated at 30°C. Isolates *Se*626 ( *S. entomophila* ) and *Se*AGR96X ( *S. proteamaculans* ) were used as the standard controls, and *Se*5.6 (pADAP) as the transconjugant control. Based on their potential role in virulence, a range of plate assays were assessed targeting extracellular enzymes lipase, protease and chitinase that are often chromosomally encoded and implicated in the degradation of insect host structural components. In this context, the ability to degrade chitin is a documented mechanism enabling the ingress of entomopathogens from the gut to the haemocoel (Son et al. 2024). Chitinase plates were prepared using a medium from Hsu and Lockwood (1975). DNase plates were used to assess the production of extracellular DNase production, known to aid in the restriction of foreign DNA acquisition in some pathogens (Blokesch and Schoolnik 2008). Assays for protease (Morris et al. 2012), lipase (Kumar et al. 2012) were performed as described to identify the role of the chromosome in degrading host tissue. Phosphatase activity was assessed as per Pikovskaya and Pikovskaya (1948) due to its role in disruption of key host cell functions. Finally, Salkowski's colorimetric test for IAA quantification (Donati et al. 2013) was used to determine the ability of *Serratia* to stimulate plant growth, which may reflect a potential plant association ability niche of the tested isolate as grass grub and manuka beetle larvae feed in the plant root zone.

### 2.4 DNA Preparation and Sequencing

Standard molecular techniques were undertaken as outlined by Green and Sambrook (2012). DNA extractions were performed using the Bioline ISOLATE II Genomic DNA kit (Meridian Bioscience, UK) and Roche platinum *taq* DNA polymerase for PCR amplification following the manufacturer's instructions. Plasmid vector DNA and PCR amplicons were purified using either the Roche high pure plasmid isolation kit or the Roche high pure PCR product purification kits, respectively (Roche Diagnostics GmbH, Mannheim, Germany). Yield and purity were determined using agarose gel electrophoresis and the NanoDrop 2000 Spectrophotometer (Thermo Scientific).

Illumina DNA sequencing was performed by Macrogen Sequencing Service (South Korea). FastQC (Andrews 2010) was used to check the quality of raw sequencing reads. A5 assembly pipeline (Tritt et al. 2012) was used to assemble whole prokaryote genomes, where contigs were trimmed using circulator (Hunt et al. 2015) to remove overhangs in circular DNA assemblies and plasmid sequences (if present). Scaffolds of *S. entomophila* genomes were mapped to isolate *Se*626 (Accession: CP074347). CheckM (Parks et al. 2015) was used to assess the quality of the microbial genome, with high genome completeness (*Se*477 = 99.9%, *Sp*3041 = 99.98%, *Se*iDIA = 99.9%) and no sequence contamination recorded (< 1%). Genome annotation was performed by PROKKA Rapid Prokaryotic Genome Annotation software (Seemann 2014).

### 2.5 Bioassays

Field collected larvae are variable in their susceptibility to pathogens which can lead to differences between bioassay results. To reduce variability healthy third instar *C. giveni* were pre‐fed for 3 days on freshly cut carrot cube (3–4 mm^3^), enabling the screening for disease‐free larvae suitable for bioassay as outlined by Hurst et al. (2018). Healthy larvae (denoted as consuming all the carrot cube and of a grey colouration) were then provided with carrot (3–4 mm^3^ in size) that had been rolled on bacterial lawn of a selected bacterial inoculum grown on LB agar plates incubated overnight at 30°C (approximately 1 × 10^7^ CFU per larvae). Each treatment comprised two replicates of six larvae. The larvae were transferred to clean trays containing fresh, untreated carrot cubes on days three and six. Uninoculated carrot was used as the negative control, with positive controls comprising treatments with either *Se*A1MO2 or *Sp*AGR96X. Symptoms of disease (non‐feeding, amber discolouration) were visually assessed on Days 3, 6, 9 and 12.

LC_50_/LT_50_ were determined using the standard larval bioassay but instead using a bacterial inoculum and concentrations derived from serial dilutions. For each dilution, 5 μL was pipetted per carrot cube. LC_50_/LT_50_ were calculated using Probit analysis:

𝑃=α+[𝑙𝑜𝑔10(𝐷𝑜𝑠𝑒)]

For LC_50_, 12 disease observations for each dilution on Day 12 were converted to the proportion of disease to healthy and corrected for control mortality prior analysis. Bioassays were omitted if > 25% disease was observed in the uninoculated control. The proportion of disease present at each time point was used for LT_50_. Bioassays were undertaken in triplicate, and the averages are presented with the standard error of the mean (𝑆𝐸 = 𝜎 √𝑛).

### 2.6 Enumeration of Bacteria From Larval Macerates

To validate the presence and identity of the pathogen from challenged larvae, bacteria were isolated from individuals exhibiting similar phenotypes that differed in disease progression between the assessment days. Individual larvae were weighed before macerating in a total volume of 1 mL dd.H_2_0. Larval macerates at days 3, 6, 9 and 12 (three replicates per time point) were subjected to serial dilution and plated on LB agar containing the appropriate antibiotics selective for the isolated strain, CTA plate selection for *Serratia* spp. and itaconate plates to select specifically for *S. entomophila* . Prospective *S. entomophila* isolates were further validated by patching colonies onto DNase and adonitol media plates (O'Callaghan and Jackson 1993) and through BOXA1R PCR. To determine whether there was any plasmid loss, Day 12 samples were patched from CTA onto plates with antibiotics selective for the plasmid tag.

### 2.7 RNA Sample Preparation

RNA was prepared from triplicate 50 mL LB broth cultures containing 2–5 × 10^9^ CFUs/mL of selected bacteria (Table 1). Initially, 3 mL overnight LB broth cultures were prepared with appropriate antibiotics from which 50 μL of the overnight culture was transferred to 50 mL LB broth without antibiotics and shaken at 30°C at 250 rpm in a Ratek orbital incubator.

RNAprotect Bacterial Reagent (Qiagen, Germany) was added in a 5:1 ratio following the manufacturer's instructions. RNA isolation was then performed using the RNA mini kit (Qiagen) following the manufacturer's instructions. Following an on‐column DNA digest, an off‐column DNA digest was also performed before the RNA mini kit clean‐up protocol. RNA samples were then precipitated overnight in isopropanol. The samples were then centrifuged (10,000*g* for 5 min at 4°C) and the resultant pellets air‐dried at 37°C for 30 mins. An aliquot of 15 μL of RNase‐free water was then added and the pellets resuspended prior to their quantification using NanoDrop 2000 Spectrophotometer and Qubit. The sample, at a concentration of 6 mg/μL, was then added to RNAstable tube kit (Sigma‐Aldrich), and liquid evaporated in a SpeedVac. Quality control was performed by Macrogen before sequencing at their facility (South Korea).

### 2.8 RNAseq

Initial quality checking of sequencing reads was undertaken using FASTQC (Andrews 2010), and the reads were then trimmed using Trimmomatic (Bolger et al. 2014). The GFF files generated from Roary annotation of *Serratia* isolate *Se*477 and *Se*A1M02 were used as a scaffold to align the short‐read libraries using HISAT2 (Kim et al. 2019), using default parameters. StringTie (Pertea et al. 2015) was used to assemble transcripts, with transcript counts calculated using BALLGOWN (Frazee et al. 2015). Differential expression was calculated using DeSeq2 (Love et al. 2014). To assess plasmid‐based virulence factor expression, transcript IDs for *Se*A1M02 and transconjugant *Se*477 (pADAP) were compared using the plasmid annotations of *Se*A1M02 (accession: NC_002523.5).

### 2.9 Statistical Analysis

*p*‐Values were generated using a two‐sample *t*‐test for bioassay data based on the instance of disease, death or combined outcome relative to the untreated control for each assay. For plate assays, Tukey's range test and general linear models were conducted in Minitab 18 to determine differences in enzymatic activity between transconjugants. Error bars for technical replicates used in graphs of bioassay data and infectivity assays generated in GraphPad Prism 9.2 and R Studio were generated as the standard error of the mean.

## 3 Results

### 3.1 Accessory Determinants and Plant Beneficial Traits

**FIGURE 1:**
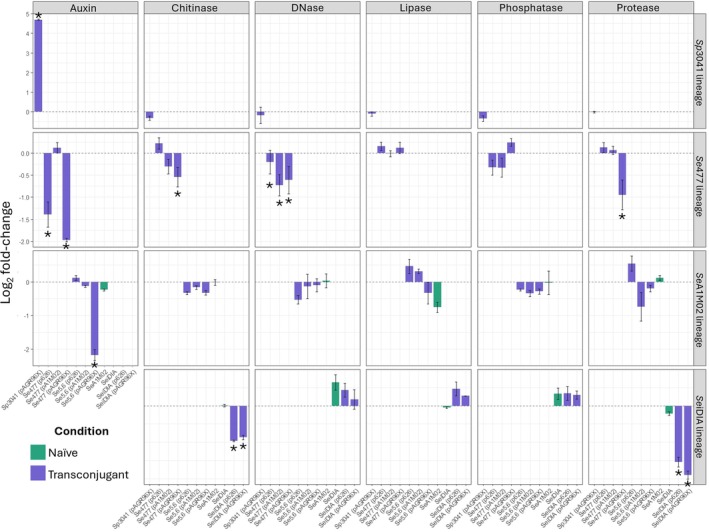
Lineage‐specific effects of plasmid carriage on enzyme activity (mean ± standard error, log2 fold‐change vs. plasmid‐free reference). Each panel shows the mean log2 fold‐change in enzyme activity relative to the plasmid‐free strain of that lineage (i.e., *Se*5.6, *Se*477, *Sp*3041 and *Se*iDIA^−^; dashed line = 0 = reference). Bars indicate mean ± standard error across biological replicates. Positive values indicate increased activity in plasmid‐bearing strains, while negative values indicate reduced activity relative to the lineage's plasmid‐free control. Rows correspond to distinct *Serratia* lineages, and columns to different enzyme assays. Colours denote experimental conditions: Blue = transconjugant, green = naïve strain (only for lineages where the naïve strain is not plasmid‐free). Asterisk denotes strains with statistically significant production of enzymes relative to the plasmid‐free strain. Additional plots of halo ratios are found in Figures S1 and S2.

**FIGURE 2:**
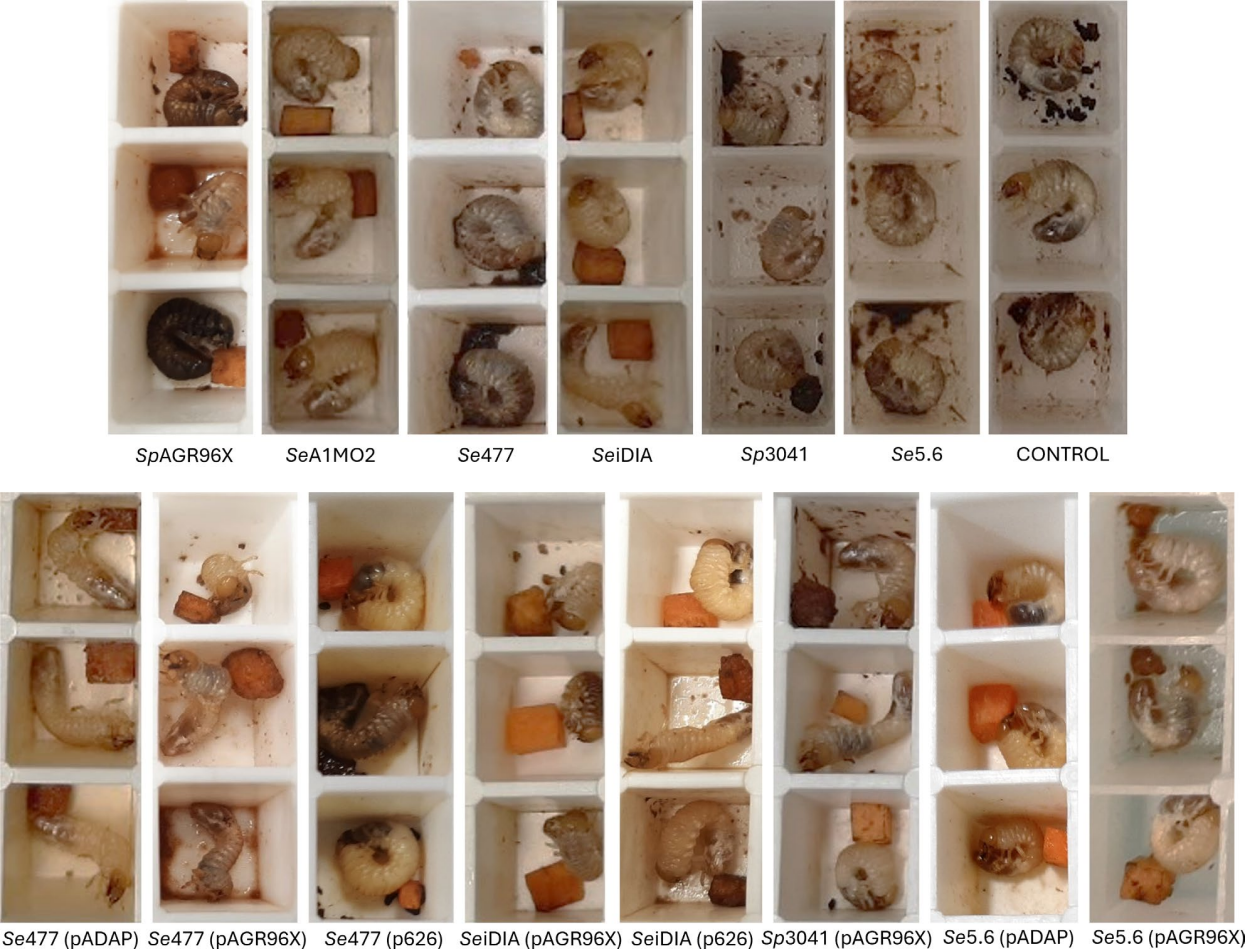
Photographs of disease phenologies of transconjugants and their naïve strain counterparts at day seven. Top row shows the naïve strain and the bottom the transconjugant plasmid derivatives. Cessation of feeding was recorded where the carrot cubes had no sign of consumption. Amber discolouration due to amber disease derived gut clearance.

Plate assays were used to assess the production of chromosomally encoded secondary virulence factors associated with host colonisation and pathogenicity to evaluate how plasmid acquisition influenced the expression of these beneficial traits. Here, we used the transconjugant *Se*5.6 (pADAP) as a control, due to it being a restoration of the original pADAP variant to the plasmid cured strain *Se*5.6. This strain exhibited the least amount of phenotypic change in comparison to *Se*5.6 (Figure 1), suggesting a tight relationship between the chromosome and its plasmid. This also validated that the removal and subsequent reintroduction of pADAP into the same cell does not impact the production of these accessory virulence factors. By using various enzyme‐based assays selected to quantify enzymatic activity associate with enhanced pathogenicity (Figure 1), the *Se*477 transconjugants *Se*477 (pAGR96X) had a significant reduction in protease production (*p* = 021) and chitinase degradation (*p* = 0.003) (Figure 1). In contrast, *Se*477 (pADAP or p626) transconjugants had a negligible effect on the production of all enzymes relative to *Se*477. Relative to *Se*477, its transconjugants (p626, pADAP or pAGR96X) had significantly reduced DNase production (*p* = 0.025) (Figure 1). Within proteolytic plate assays, the transconjugant *Se*iDIA (pAGR96X) showed significantly reduced proteolytic activity relative to their respective naïve counterparts (*p* = 0.042) (Figure 1). Both *Se*iDIA (pAGR96X) and *Se*iDIA (p626) exhibited significantly reduced (*p* < 0.01 for both cases) chitinase activity relative to *Se*iDIA (Figure 2). Exemplar plates and halo to colony ratios are shown in Figures S1 and S2.

Through IAA quantification assays, the *Se*477 (pAGR96X), *Se*5.6 (pAGR96X) and *Se*iDIA (pAGR96X) transconjugants were shown to have reduced auxin production, while *Sp*3041 (pAGR96X) transconjugants exhibited increased auxin activity (Figure 1). In silico analysis of the *Sp*AGR96X and *Sp*3041 genomes revealed that these isolates encoded two or three copies of the decarboxylation gene *ipdC*, the product of which converts indole‐3‐pyruvic acid to indole‐3‐acetaldehyde, step of the tryptophan dependent IAA biosynthetic pathway. In contrast, *S. entomophilia* strains *Se*477 and *Se*iDIA encode a single *ipdC* copy.

### 3.2 LC_50_ and LT_50_ of *Serratia*‐Challenged Grass Grub Larvae and Plasmid Stability

Based on the altered plate assay phenotype of transconjugants, we sought to determine if the virulence capacity of the transconjugants relative to their naïve strain was also altered. LC_50_ and LT_50_ bioassays were conducted comparing transconjugants with naïve strains that were either plasmid‐bearing or plasmid‐free. This approach allowed us to assess whether exchanging plasmids between hosts altered the level of conferred pathogenicity, distinguishing effects that were either driven by the intrinsic virulence potential of that plasmid (resulting in similar results to the donor host) or by its interaction with the host chromosome (significantly variable to the donor host) (Figure 2). Consistent with results from enzymatic plate assays, *C. giveni* larvae challenged with *Se*5.6 (pADAP) transconjugant exhibited the same disease pathotype as *Se*A1MO2 after approximately 4 days, while in *Se*5.6 (p626) amber discolouration was observed alongside a non‐uniform cessation of feeding at day four (Table 3; Figure 2). The *Se*477 (p626) and *Se*477 (pADAP) transconjugants exhibited similar disease levels to *Se*A1MO2 or *Se*626 donor with consistent amber colouration observed across each of the challenged larvae (Figure 2; Table 3).

**TABLE 3:** Percentage of challenged larvae exhibiting disease at Day 12.

Isolate	LC_50_ ^a^ (±SE)	LT_50_ ^b^ (days)	Diseased (%) ± SE	Dead (%) ± SE	Combined diseased/dead (%) ± SE	Pathotype
Untreated	**—**	**—**	0 ± 0	30.56 ± 7.78	30.56 ± 7.78	—
*Se*5.6	**—**	**—**	**28.57 ± 10.10**	9.52 ± 6.56	38.1 ± 10.85	Non‐path
*Se*477	**—**	**—**	5 ± 5	15 ± 8.19	20 ± 9.17	Non‐path
*Sp*3041	**—**	**—**	6.06 ± 4.21	24.24 ± 7.57	30.3 ± 8.12	Non‐path
*Se*626	2.6 × 10^−5^ (±1.9 × 10^−5^)	4	**71.43 ± 10.10**	28.57 ± 10.10	**100 ± 0**	Pathogenic
*Se*A1M02	2.9 × 10^−5^ (±2.7 × 10^−5^)	3	**71.43 ± 10.10**	28.57 ± 10.10	**100 ± 0**	Pathogenic
*Sp*AGR96X	4.9 × 10^−4^ (±3.6 × 10^−4^)	3	**42.86 ± 11.06**	52.38 ± 11.16	**95.24 ± 4.76**	Hyper virulent
*Se*iDIA	2.04 × 10^−4^ (±1.5 × 10^−2^)	4	**62.5 ± 8.69**	28.13 ± 8.07	**90.63 ± 5.23**	Pathogenic
*Sp*3041 (pAGR96X)	1.9 × 10^−6^ (±1.5 × 10^−6^)	7	**36.36 ± 8.50**	33.33 ± 8.33	**69.7 ± 8.12**	Pathogenic
*Se*477 (p626)	8.6 × 10^−5^ (±8.5 × 10^−5^)	4	**72.73 ± 7.87**	27.27 ± 7.87	**100 ± 0**	Pathogenic
*Se*477 (pADAP)	4.9 × 10^−5^ (±2.4 × 10^−5^)	4	**54.55 ± 8.80**	36.36 ± 8.50	**90.91 ± 5.08**	Pathogenic
*Se*477 (pAGR96X)	6.8 × 10^−6^ (±6.8 × 10^−6^)	4	**60.61 ± 8.63**	21.21 ± 7.22	**81.82 ± 6.81**	Variable
*Se*5.6 (p626)	5.3 × 10^−7^ (±5.3 × 10^−7^)	4	**28.57 ± 10.10**	33.33 ± 10.54	**61.9 ± 10.85**	Variable
*Se*5.6 (pADAP)	6.4 × 10^−6^ (±6.4 × 10^−6^)	4	**72.73 ± 7.87**	27.27 ± 7.87	**100 ± 0**	Pathogenic
*Se*5.6 (pAGR96X)	1.08 × 10^−7^ (±1.01 × 10^−7^)	3	**27.27 ± 7.87**	18.18 ± 6.81	45.45 ± 8.80	Variable
*Se*iDIA (p626)	2.2 × 10^−4^ (±1.1 × 10^−4^)	4	**78.79 ± 7.22**	21.21 ± 7.22	**100 ± 0**	Pathogenic
*Se*iDIA (pAGR96X)	7.7 × 10^−5^ (±7.1 × 10^−5^)	7	**36.36 ± 8.50**	12.12 ± 5.76	48.48 ± 8.83	Variable

*Note: p*‐Values (Fisher's exact), with significance in relation to the negative control (*p* < 0.05), highlighted in bold.

^a^ Undertaken via probit analysis.

^b^ Statistical survival analysis.

Bioassays of *C. giveni* larvae challenged with the *Se*5.6 (pAGR96X), *Se*iDIA (pAGR96X), *Se*477 (pAGR96X) and *Sp*3041 (pAGR96X) transconjugants afforded inconsistent results, with only a proportion of the observed larvae succumbing to disease. Although the *Se*477 (pAGR96X) and *Sp*3041 (pAGR96X) transconjugants conferred virulence, their activity was reduced relative to *Sp*AGR96X. The LC_50_ of the *Se*477 (pAGR96X) transconjugant was significantly higher (6.8 × 10^−6^ ± 6.8 × 10^−6^ CFUs) than that of *Sp*AGR96X (4.9 × 10^−4^ ± 3.6 × 10^−4^ CFUs; Table 3), corresponding to a ~70‐fold decrease in toxicity indicating that a higher dose is required to achieve 50% mortality. The *Se*5.6 (pAGR96X) transconjugant exerted delayed phenotypic symptoms to *Sp*AGR96X, where disease was not apparent until day 3. There was no significant difference between the combined disease and mortality rates of transconjugant *Se*5.6 (pAGR96X) or *Se*iDIA (pAGR96X) and the negative control, influenced by the low mortality rates of diseased larvae in these strains and demonstrating reduced virulence compared to other transconjugants. *Se*iDIA (pAGR96X) exhibited reduced mortality and LT_50_ compared to both *Sp*AGR96X and *Se*iDIA (Table 3).

### 3.3 Quantifying Larval Bacterial Load and Plasmid Stability

**FIGURE 3:**
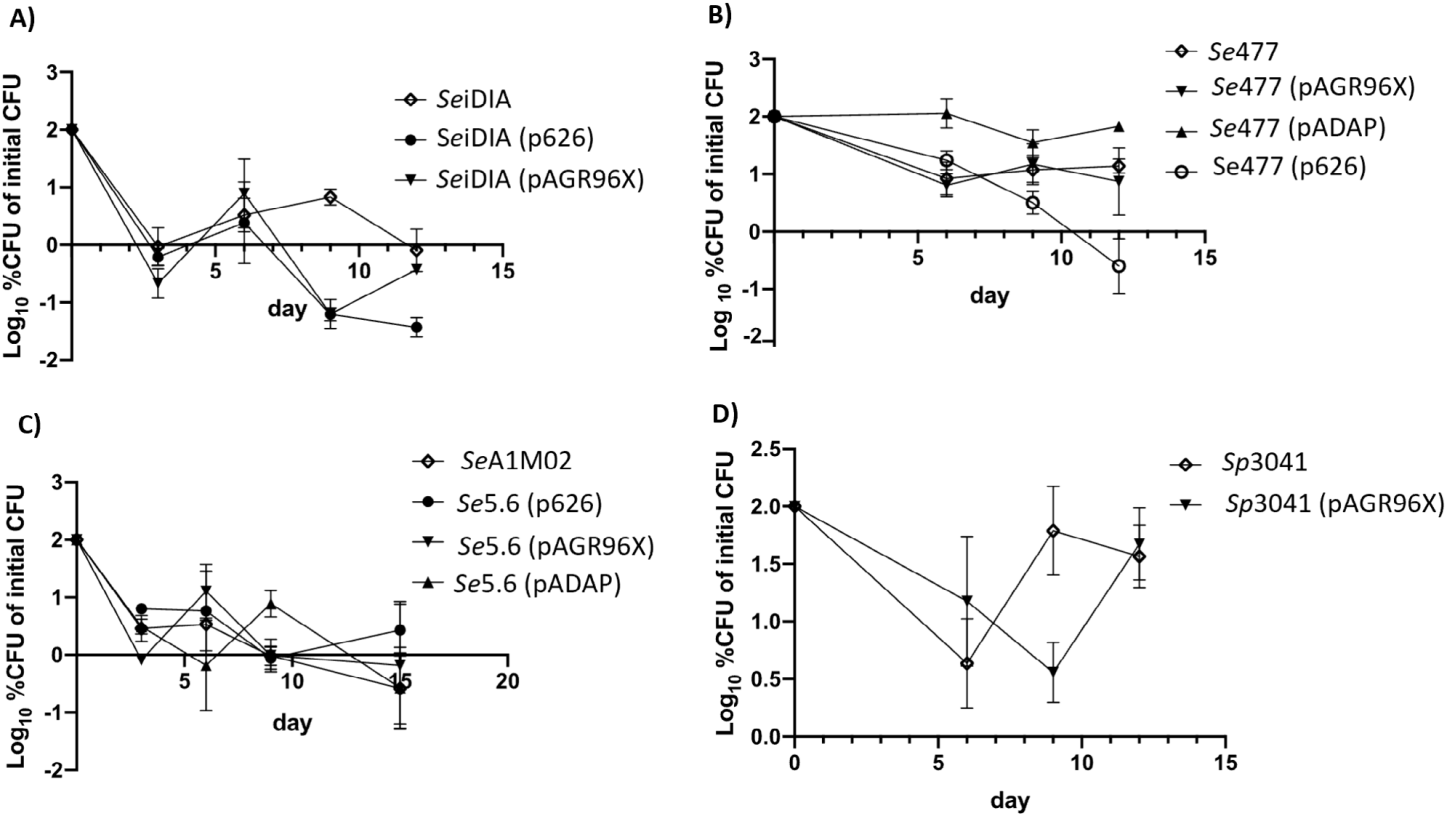
CFUs recovered from larval macerates challenged with transconjugants over a 12‐day period. (A) Plasmid transconjugants into *Se*iDIA; (B) *Se*477; (C) *Se*5.6 and (D) *Sp*3041. Data were normalised and represented as log_10_ transformed percentage of the initial CFU. Standard error bars of the means are shown.

To determine if the variability of observed pathotypes may reflect the loss of the donor plasmid over time, we assessed the bacterial cell loading of *C. giveni*‐challenged larvae at days 3, 6, 8 and 12 through the infection process (Figure 3). Enumeration of *Se*iDIA cell numbers from challenged *C. giveni* larvae showed increased bacterial CFUs at Day 12 (8.67 × 10^5^ CFU) compared to either p626 (4.3 × 10^4^ CFU) or pAGR96X (3.6 × 10^5^ CFU) transconjugants, representing an approximately 20‐fold increase relative to *Se*iDIA (p626) (Figure 3A; Table S1). Normalisation of the data showed this change in CFU not to be significant (p626 *p* = 0.081 and pAGR96X *p* = 0.465; Figure 3A).

Similarly, the number of *Se*477 (pAGR96X; 1.0 × 10^6^ CFU) isolated at 12 days was approximately 15‐fold lower than *Se*477 (1.53 × 10^7^ CFU) (Figure 3B). A *t*‐test of the transformed data at day 12 found no significant difference in CFUs between *Se*477 and its p626 and pADAP plasmid transconjugants, although CFUs of *Se*477 (p626) were lower. Similar CFUs of *Se*A1M02 or *Se*5.6 (pADAP) were recovered from the challenged larvae (Figure 3C; Table S1). Similar numbers of *Sp*3041 (4.97 × 10^7^) and *Sp*3041 (pAGR96X) (3.78 × 10^7^) cells were re‐isolated from challenged larvae (Figure 3D). At Day 12, 100% of the assessed strains (*Se*477, *Se*iDIA, *Se*A1M02 and *Sp*3041; and their transconjugants) were found to have retained the plasmid.

### 3.4 In Vitro Assessments of Wildtype and Transconjugant Fitness in Liquid Culture

Through the process of dilution plating of various transconjugants in this study, the pAGR96X transconjugants, *Se*477 (pAGR96X) and *Sp*3041 (pAGR96X), had smaller relative colony sizes than *Se*477, *Sp*3041 or the other *Se* transconjugants (Figure S3). This phenomenon also occurred on non‐selective media, suggesting potential impacts on growth in transconjugants. To determine the potential metabolic burden of non‐native plasmids imposed on the recipient strain, growth curves of *Se*477, *Se*iDIA, *Sp*3041, *Se*5.6 and their pADAP, pAGR96X and p626 plasmid transconjugants were undertaken in LB and M9 minimal media in the absence of antibiotics. Using LB broth, a slight difference in growth was observed between each naïve strain and its transconjugants. In *Se*5.6 and *Se*477, plasmid carriage caused a slight reduction in growth, visible at the stationary phase. The inverse was noted in *Se*iDIA, where overall bacterial growth was highest in the transconjugant *Se*iDIA (p626). In M9 medium, *Se*iDIA entered the exponential growth phase earlier than transconjugant *Se*iDIA (p626) and *Se*iDIA (pAGR96X), suggesting the native plasmid confers the most benefit under stress conditions. The initial growth of the transconjugants *Se*477 (p626, pADAP or pAGR96X) was observed to reach the exponential phase approximately 5 h faster than *Se*477. By 24 hpi, *Se*477 had overtaken *Se*477 (pAGR96X) in comparative OD_600_, but not compared to the *Se*477 (p626 or pADAP) transconjugants. This was the inverse trend that was observed with *Sp*3041, where *Sp*3041 was shown to outperform *Sp*3041 (pAGR96X) at all stages of growth. In M9 minimal medium, relative to *Se*5.6, the growth rate of the *Se*5.6 (p626) transconjugant was reduced, but this was not observed in LB broth (Figures S4–S7). The naïve strain *Se*A1M02 outperformed all other *Se*5.6 plasmid variants, including the restored *Se*5.6 (pADAP) transconjugant, suggesting that the original plasmid composition was optimal for growth.

### 3.5 Transcriptome Analysis

**FIGURE 4:**
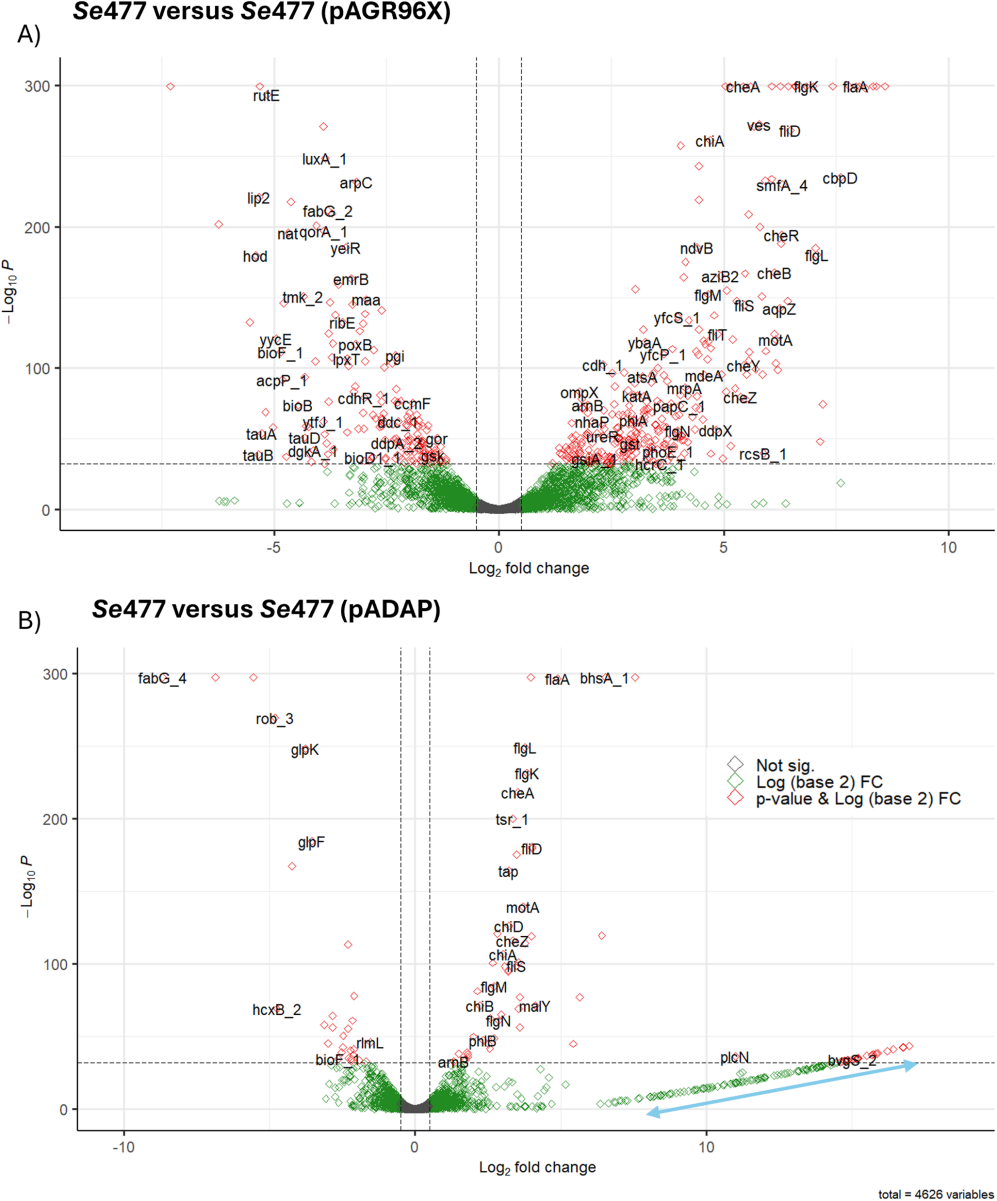
Volcano plots showing differentially expressed transcripts significantly regulated with adjusted *p*‐value (*p*_adjust_) <0.005 for chromosomally encoded genes. (A) Between *Se*477 and *Se*477 (pAGR96X); (B) between *Se*477 and *Se*477 (pADAP). Red points denote genes with significant changes in expression with an adjusted *p*‐value cut of 0.05 log2 fold change > 2; green points denote significantly differentially expressed genes with log2 fold change < 2. The cut‐off lines represent genes with relatively high expression in one condition compared to the other (where zero or close values are found). Blue arrow indicates nil expression of genomic island genes (Table S2; Figure S8).

Based on the aberrant plate assays, larval bioassay profiles and reduced fitness, *Se*477 and its transconjugants *Se*477 (pADAP) and *Se*477 (pAGR96X) were further examined through transcriptomics. Gene expression levels were determined at the stationary growth phase at 7.2 × 10^9^ CFU/mL for *Se*477, 6.1 × 10^9^ CFU/mL for *Se*477 (pADAP and p626), 6.8 × 10^9^ CFU/mL for *Se*477 (pAGR96X) and 7.5 × 10^9^ CFU/mL for *Se*A1M02. The transconjugant *Se*477 (pAGR96X) (Figure 4A) had an increased differential expression rate (~2.35‐fold increase) of chromosomal genes compared to that of the *Se*477 (pADAP) transconjugant (Figure 4B); 57 upregulated genes and 229 down regulated to log2 fold in *Se*477 pADAP; 206 upregulated genes and 466 down regulated to log2 fold in *Se*477 (pAGR96X) implicating the addition of pAGR96X relative to pADAP in altering the transcriptome profile of *Se*477 (Supporting Information File S1‐DEGS). Through comparison of *Se*477 vs. *Se*477 (pADAP) 134 transcripts showed a zero counts artefact across replicates relative to *Se*477 (Figure 4B, blue arrow). Based on the comparison of these genes with the output of *Se*477 in IslandViewer4 (Bertelli et al. 2017), 66% of these genes are associated with four predicted genomic islands (57.2, 34.3, 24.4 and 9.8 kb in size; Figure S8; Table S2). Genes co‐located on these putative islands included a restriction modification system (*hsdR* and *hsdM*) orthologs that reside on a pathogenicity island in *Vibrio cholerae* (Jermyn and Boyd 2002) and a *hipA* toxin complex of a tox/antitox system associated with a genomic island in *Shewanella putrefaciens* (Zhao et al. 2022) (Table S2). While this pattern could partially reflect mapping artefacts due to sequence composition, the consistent absence of transcripts across replicates suggests genuine transcriptional silencing of this region.

**FIGURE 5:**
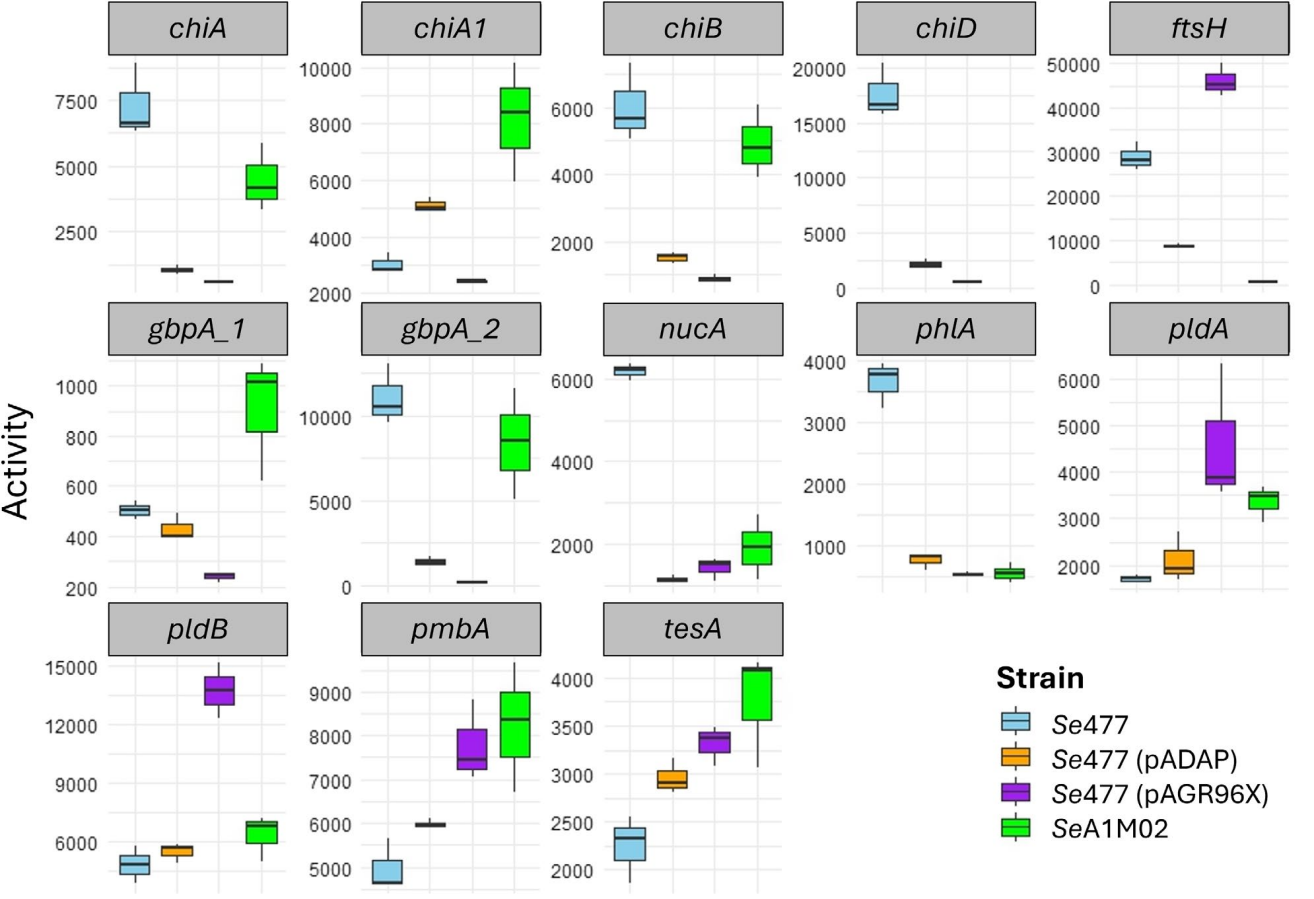
Transcriptome reads count of genes associated with virulence factor production transconjugants *Se*477 (pAGR96X) and *Se*477 (pADAP) compared to *Se*477 and *Se*A1M02. Read counts in triplicate. Error bars indicate standard error of the mean.

We then sought to define any correlation between transcriptome profiles and the phenotypes observed in the plate assays. Transcription of the *nucA* endonuclease gene associated with DNase production was significantly downregulated (log2 fold change > 2, *p* < 0.05) in both transconjugants *Se*477 (pADAP) and *Se*477 (pAGR96X) relative to *Se*477 (Figure 5). Like *Se*477 (pADAP) a low level of *nucA* expression was noted in *Se*A1M02. Relative to *Se*477, the transcriptional regulator *nucC* was downregulated in *Se*477 (pADAP) but did not significantly differ in *Se*477 (pAGR96X), data that correlated with the DNase plate assay (Figure 1).

In both *Se*477 (pAGR96X and pADAP) transconjugants, the chitinase encoding genes *chiA, chiB* and *chiD* (*chiD* is absent in *Se*A1M02 and *Sei*DIA) were downregulated to log2 fold > 2 relative to *Se*477 (Figure 5). The expression of the *Se*477 (pAGR96X) *chiA* gene was significantly downregulated to log2 fold < 2, while the *Se*477 (pADAP), *chiA1* was upregulated to log2 fold < 2 relative to *Se*477. The expression of *Se*477 *chiA, chiD* and *chiB* was log2 fold < 2 significantly upregulated relative to the transconjugant *Se*477 (pADAP). This concurs with plate assays where the plasmid transconjugants *Se*477 (pAGR96X), *Se*477 (pADAP), *Se*iDIA (p626), *Se*iDIA (pAGR96X) exhibited significantly reduced chitinolytic activity relative to *Se*477 (Figure 1).

Transcriptome analysis of the *gbpA_1* chitin binding protein showed no significant difference between *Se*477 and *Se*477 (pADAP), while log2 fold change < 2 of *gbpA_1* was observed in *Se*477 (pAGR96X) (Figure 5). The expression of chitin‐binding protein *gbpA_1* in *Se*477 (pADAP) did not differ from *Se*A1M02. However, for both Se477 plasmid transconjugants, the expression of *gbpA_2* was downregulated (log2 fold change < 2). Significant differentiation of expression was also observed between *Se*477 (pADAP) and *Se*A1M02 (log2 fold < 2 for *gbpA_1* and log2 fold > 2 for *gbpA_2*).

Transcriptome assessments of the four phospholipase genes identified through in silico genome analysis identified differential expression between the *Se*477 (pADAP) and (pAGR96X) transconjugants in *phlA* (extracellular phospholipase 1), *pldA*, *pldB* (phospholipase A1 and B) and *tesA* (lysophospholipase L1). In both transconjugants, the production of extracellular phospholipase A1 was downregulated relative to *Se*477 (Figure 5). In *Se*477 (pADAP), though the *tesA* gene was significantly upregulated (log2 fold < 2), neither *pldA* nor *pldB* showed any significant differentiation of expression. In *Se*477 (pAGR96X), *tesA, pldA* and *pldB* showed a significant upregulation to log2 fold < 2 compared to *Se*477. This contrasted to plate assays, which, although they showed an increased production of lipases in *Se*477 (pAGR96X), showed no significant difference in lipolytic activity. An additional comparison of *Se*A1M02 with *Se*477 (pADAP) transconjugant revealed a significance of differentiated gene expression (log2 fold < 2) in the upregula9tion of *pldA* in *Se*A1M02, with no significant difference in gene expression observed between *tesA, phlA* and *pldB*.

Relative to *Se*477, the ATP‐dependent zinc‐metalloprotease *ftsH* and the metalloprotease *pmbA* were upregulated in *Se*477 (pAGR96X) (log2 fold change < 2). In *Se*477, *ftsH* was upregulated (log2 fold > 2) relative to transconjugant *Se*477 (pADAP), whereas no significant difference was observed with *pmbA*.

### 3.6 Expression Profile of Plasmid‐Based Virulence Determinants

**FIGURE 6:**
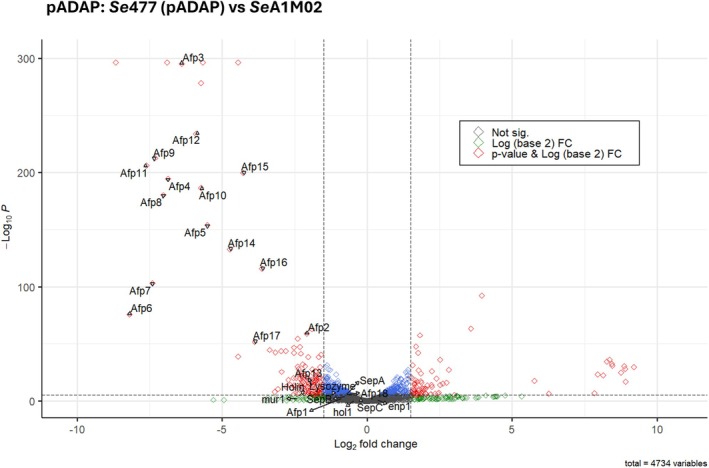
Volcano plot showing differentially expressed virulence‐related transcripts significantly regulated with *p*_adjust_ < 0.005 between plasmid‐associated transcripts between *Se*A1M02 and *Se*477 (pADAP). Red points denote genes with significant changes in expression with an adjusted *p*‐value cut off at 0.05, log2 fold change > 2; green points denote significant differentially expressed genes with log2 fold change < 2. The cutoff lines represent genes with relatively high expression in one condition compared to the other (where zero or close values are found). Labels are added for genes associated with the Afp and Sep Tcs.

Transcriptome assessment of *Se*A1M02 and *Se*477 (pADAP) revealed a log2 fold increase in the pADAP‐encoded *afp* and *sep* Tc virulence clusters (Table 4, Figure 6). In *Se*477 (pADAP), most *afp1‐18* genes were upregulated, showing log2 fold changes > 2 (Table 4; Figure 6). The expression of the predicted Afp lysis gene *mur*1, located immediately upstream of *afp1* of *Se*A1MO2, was log2 fold > 2 differentiation of higher gene expression relative to the *Se*477 (pADAP). In addition, relative to the *Se*A1M02, within *Se*477 (pADAP) there was significant upregulation of *sepA* and *sepB* (log2 fold > 2), but not the associated *sepC;* the expression of the lysozyme and holin 5′ of the *sepA* gene also showed a log2 fold > 2 increase.

**TABLE 4:** Differential expression of Afp and Sep encoding genes between transconjugant *Se*477 (pADAP) and *Se*A1MO2.

Transcript ID	Annotation	*Se*477 (pADAP)	*Se*A1M02	LOG2FC
JJLEJPGA_03779	Holin	137 ± 11.26	36 ± 11.53	**−2.17**
JJLEJPGA_03780	Lysozyme	213.33 ± 2.09	385.66 ± 80.82	−1.15
JJLEJPGA_03781	*sepA*	11,463.67 ± 1133.52	8613.66 ± 2300.83	−0.72
JJLEJPGA_03782	*sepB*	7549 ± 775.10	5554.66 ± 1354.21	−0.74
JJLEJPGA_03784	*sepC*	7684.33 ± 468.01	7879 ± 1746.36	−0.27
JJLEJPGA_03789	*enp1*	317 ± 41.72	486 ± 2.14	0.33
JJLEJPGA_03790	*hol1*	58 ± 23.43	51.33 ± 25.10	−0.46
JJLEJPGA_03791	*mur1*	23 ± 6.56	5.67 ± 4.93	**−2.4**
JJLEJPGA_03792	*afp1*	119.67 ± 16.77	81 ± 15.62	−0.86
JJLEJPGA_03793	*afp2*	2538.66 ± 223.28	746 ± 165.50	**−2.07**
JJLEJPGA_03794	*afp3*	82,462.33 ± 14,421.19	1213 ± 302.22	**−6.39**
JJLEJPGA_03795	*afp4*	54,530.33 ± 12,685.73	579 ± 209.06	**−6.86**
JJLEJPGA_03796	*afp5*	6396.66 ± 1471.4	171 ± 16.09	**−5.5**
JJLEJPGA_03797	*afp6*	1346 ± 397.05	5.66 ± 4.16	**−8.18**
JJLEJPGA_03798	*afp7*	11,142 ± 3849.99	79 ± 32.92	**−7.4**
JJLEJPGA_03799	*afp8*	29,071 ± 11,092.33	276 ± 54.28	**−7.02**
JJLEJPGA_03800	*afp9*	14,806 ± 3998.46	112.66 ± 4.93	**−7.3**
JJLEJPGA_03801	*afp10*	17,787.67 ± 4123.50	418.66 ± 120.44	**−5.73**
JJLEJPGA_03802	*afp11*	60,666.33 ± 16,701.89	373.66 ± 78.14	**−7.61**
JJLEJPGA_03803	*afp12*	83,096.67 ± 19,656.25	1716 ± 338.51	**−5.89**
JJLEJPGA_03804	*afp13*	681.6667 ± 50.50	218.66 ± 24.94	−1.9
JJLEJPGA_04489	*afp14*	85,543.67 ± 5141.35	90,931.33 ± 19,820.70	**−4.73**
JJLEJPGA_04490	*afp15*	8875.667 ± 489.60	560.3333 ± 81.98	**−4.28**
JJLEJPGA_04491	*afp16*	2239 ± 244.69	222.6667 ± 31.66	**−3.62**
JJLEJPGA_04492	*afp17*	1096 ± 212.58	93.33333 ± 32.08	**−3.87**
JJLEJPGA_04493	*afp18*	7421 ± 382.51	5505.333 ± 495.19	**−0.72**

*Note:* Annotations from pADAP from GenBank NC_002523.5. Bolded shows *p* < 0.01, log2 fold > 2. Underlined shows *p* < 0.05, log2 fold > 2.

## 4 Discussion

Conjugative plasmids are known to drive bacterial evolution, such as the horizontally acquired plasmids that confer pathogenicity in *Clostridium perfringens* (Watts et al. 2022) and those that increase the spread of antibiotic resistance in *E. coli* (Palomino et al. 2023). Unravelling the limits and fitness costs incurred by plasmid transfer is key to understanding the plasmid paradox, the persistence of plasmids in bacterial populations despite the fitness costs they often impose (Brockhurst and Harrison 2022), and its role in evolution. Our work suggests that not only do plasmids persist because of their benefits to host bacteria, but also by mechanisms of host‐plasmid specificity, reducing the net metabolic fitness burden—a crucial step in resolving this paradox.

Through the use of transcriptomics and phenotypic assays, we have demonstrated that the conjugative transfer of pADAP and STAMP variants to a non‐native *Serratia* host conferred variable pathogenicity and altered the expression and production of secondary virulence factors and metabolites in recipients. The observed differences in phenotype and transcription on acquisition of a non‐native plasmid are in agreement with data of Sitter et al. (2021) and Grkovic et al. (1995), who observed variable pathotypes in transconjugant strains of *Serratia*. Similar trends have been noted within *Klebsiella pneumoniae*, where plasmid transfer reduced virulence traits such as biofilm formation of the recipient strain (Tian et al. 2021). Likewise, the recipients of the *S. proteamaculans Sp*AGR96X‐derived plasmid (pAGR96X) did not acquire its hypervirulence. However, the transconjugant *Se*iDIA (p626) shared a similar virulence profile to *Se*626 and *Se*A1M02, suggesting that the onset of disease is solely attributed to the plasmid. We note that our bioassays use field‐collected larvae that can exhibit natural variability in susceptibility to infection. While standardisation was undertaken to minimise background disease, this variability of susceptibility likely contributed to the high standard errors observed in LC_50_ estimates, a phenomenon also reported in similar studies using field populations (Miller et al. 2010; Castrejón‐Antonio and Tamez‐Guerra 2025). Conducting equivalent bioassays under controlled laboratory conditions using reared *C. giveni* larvae would help to reduce host‐related variability and provide a clearer assessment of pathogen virulence; however, laboratory rearing of this scarab is very difficult.

Previous studies utilising phenotypic assays in unison with metabolomic analysis determined that the fitness burden and benefits of plasmids are multifactorial (Dimitriu et al. 2016; Rajer and Sandegren 2022; San Millan et al. 2018). We found production of accessory virulence determinants (notably, chitinases and proteases) was altered for all the assessed transconjugants. Chitinases are well documented as key mediators that enable pathogens to gain ingress into the insect's haemocoel, leading to infection of the host (Son et al. 2024), where *S. marcescens* derived proteases and chitinases have been implicated with *Anopheles dirus* larvicidal activity (Jupatanakul et al. 2020) and chitinases of *Pseudomonas protegens* implicated in degradation of the peritrophic membrane of larvae of the diamondback moth *Plutella xylostella* (Vesga et al. 2020) and the house fly *Musca domestica* (Ruiu and Mura 2021). Here, none of the assessed transconjugants were observed to have significantly elevated chitinase production relative to their naïve strains. The transconjugants *Se*477 (pAGR96X) and *Se*iDIA (pAGR96X) exhibited reduced capacity to produce chitinases, consistent with transcriptomic data showing lower read count for *chiA*, *chiA1*, *chiB* and *chiD*. This was less pronounced on chitinolytic plate assays for *Se*477, *Se*5.6 and *Se*iDIA transconjugants (pADAP and p626) or transconjugant *Sp*3041 (pAGR96X). Relative to *Se*477, the reduced transcription of *chiD* in *Se*477 (pAGR96X) was reflected in reduced plate‐based chitinase activity. This, in conjunction with the irregular pathogenicity of the pAGR96X transconjugants, may reflect a reduced capacity of the bacterial toxins and/or bacteria to penetrate the peritrophic membrane. Genome‐based assessments of the *S. entomophila Se*A1M02 and *Sei*DIA genomes found no *chiD* to be present (unlike in *Se*477 and *Se*626), which may reflect the inability of these isolates to breach the intestine until the final stages of disease, reducing mortality relative to hyper‐virulent strains such as *Sp*AGR96X.

We found that relative to *Se*477, the phospholipase gene *pldB* was upregulated in *Se*477 (pAGR96X), as also evidenced in lipolytic plate assays. Lipolytic enzymes such as *pldB* may have a role in virulence by activating the phosphoinositide 3‐kinase (PI3K/Akt) pathway and evading the host immune system, as also demonstrated in *Pseudomonas aeruginosa* (Jiang et al. 2014). Similarly, the transconjugant *Sp*3041 (pAGR96X) had reduced and variable efficacy against challenged *C. giveni* larvae, which may reflect the aberrant synthesis of these accessory determinants. In these assays, no significant differences in the CFUs isolated from naïve strains or the transconjugants were noted, suggesting bacterial load was not a factor in irregular pathotypes.

Plasmids that have evolved with their host share a similar GC profile as their host chromosome, have few molecular alterations and have fewer fitness impacts, resulting in a lower metabolic burden and smaller changes to host transcription profiles (San Millan et al. 2018). The plasmid pADAP shares > 99.9% plasmid nucleotide identity to other pADAP variants (Sitter et al. 2021). Based on this and the absence of non‐pADAP STAMPs in *S. entomophila*, it has been proposed that *Se*A1M02 and other plasmid‐carrying strains of *Serratia* have coevolved with their plasmid to a tightly regulated mechanism of chronic disease (Hurst et al. 2023). The free‐living *S. entomophila* isolates *Se*A1M02, *Se*iDIA and *Se*477—which share a high degree of nucleotide similarity (Sitter et al. 2021; Vaughan 2021)—readily acquired pADAP plasmid types with no obvious burden to growth. In contrast, conjugation of pAGR96X to *Se*477 and *Se*iDIA yielded opposing results marked by reduced acquisition of virulence traits. The plasmid transconjugant *Se*477 (pAGR96X) exhibited reduced growth on agar plates, likely reflecting additional factors detrimental to cell growth. In support of this, transcriptomic analysis of *Se*477 (pADAP) revealed that on acquisition of pADAP, the *sepABC* (significantly for *sepA*), *afp1‐18* virulence genes (*p* < 0.05) and the associated lysis cluster (*mur1 p* < 0.01) were all upregulated. In contrast, the lysis cassette *mur1* 5′ of the Afp (Hurst et al. 2004) was significantly downregulated. In the latter instance, it is plausible that the elevated expression of Afp in *Se*477 (pADAP) would be harmful to the transconjugant strain, exhausting metabolic resources (Bolognesi and Lehner 2018).

While STAMPs can be conjugated into *S. entomophila* and *S. proteamaculans* under laboratory conditions, full plasmid‐encoded disease is not realised in all transconjugants where only a proportion of challenged larvae exhibited disease symptoms, as exemplified in the naturally plasmid‐free isolate *Se*477. This supports the hypothesis that STAMP variants and their bacterial hosts, have speciated to suit a virulence‐based lifestyle, while plasmid‐free strains favour non‐pathogenic lifestyle. Furthermore, the successful addition of plasmids from closely related chromosomal backgrounds evidenced here between *S. entomophila* strains (i.e., > 98% nucleotide identity Vaughan et al. 2022; San Millan et al. 2018) suggests that metabolic burden is reduced when fewer molecular alterations are introduced, and supports previous research on donor‐recipient compatibility (Tokuda et al. 2020). Similar mechanisms are described in the *E. coli* F‐like plasmids, where host range is mediated by four distinct isoforms of membrane protein in a recipient cell (Frankel et al. 2023), outer membrane interactions of compatible proteins for stable DNA transfer (Tokuda et al. 2020), indicative of a similarity‐based selection process.

Our results show that the presence of a non‐native plasmid alters the transcriptome and pathogenicity profile, limiting the ability of the cell to acquire full pathogenicity, potentially based on mis‐timed regulation and/or host cell overburdening. This would account for some of the reduced virulence of the assessed *Se*iDIA, *Se*477, *Se*5.6 and *Sp*3041 (pAGR96X) transconjugants against challenged grass grub larvae. Combined, these findings support the hypothesis that the chromosomal background influences plasmid‐mediated disease expression, suggesting that pathogenicity is modulated by host‐plasmid compatibility (Sitter et al. 2021). Extending from this, it is likely that environmental plasmid‐free *Serratia* variants such as *Se*477 (O'Callaghan and Jackson 1993) have always been plasmid‐free, where they are more suited to a saprophytic lifestyle than their entomopathogenic counterparts.

Based on the differing transcriptome and phenotypic profiles of the transconjugants, it is likely that gene regulation has a key role in stabilising the relationship between the bacterial chromosome and their associated plasmids, allowing the latter to persist within a lineage. Further work is necessary to define the plasmid‐based systems that alter gene regulation.

## Author Contributions

**Mark R. H. Hurst:** conceptualization, funding acquisition, project administration, resources, supervision, methodology, writing – review and editing. **Travis R. Glare:** conceptualization, funding acquistion, supervision, writing – review and editing. **Charles A. Hefer:** data curation, software, investigation, formal analysis, writing – review and editing. **Amy L. Vaughan:** investigation, writing – original draft, methodology, validation, visualization, data curation, writing – review and editing.

## Funding

This work was supported by the Tertiary Education Commission.

## Ethics Statement

The authors have nothing to report.

## Conflicts of Interest

The authors declare no conflicts of interest.

## Supporting information


**Supporting Information: File 1‐DEGS.** Complete dataset of differentially expressed across three sheets. Cells are shaded green or blue where the corresponding strain is upregulated, with an adjusted *p*‐value cut off at 0.05. Log2 fold change > 2 is represented with dark shading and light shaded cells denote significant differentially expressed genes with log2 fold change < 2. (A) Complete dataset of DEGs of chromosomal transcripts between *Se*477 and *Se*477 (pAGR96X). (B) Complete dataset of DEGs of chromosomal transcripts between *Se*477 and *Se*477 (pA1M02) and (C) Complete dataset of DEGs of plasmid‐associated transcripts between *Se*A1M02 and *Se*477 (pADAP).

**Table S1:** Number of CFUs recovered from host macerates of larvae inoculated with transconjugants and their wildtype counterparts from Days 6 to 12.

**Table S2:** Transcript data corresponding to putative genomic islands predicted for erroneous expression artefact in transcriptome data for *Se*477 versus Se477 (pADAP).

**Figure S1:** Protease and DNase enzyme halo ratios and exemplar plates. Average ratio of protease/DNase expression with standard error for all isolates split by species (red recipient chromosome *Sp*; blue recipient chromosome *Se*). Exemplar plates show a selection of naïve strains and transconjugants that were then measured.
**Figure S2:** Lipase and chitinase enzyme halo ratios and exemplar plates. Average ratio of lipase/chitinase expression with standard error for all isolates split by species (red recipient chromosome *Sp*; blue recipient chromosome *Se*). Exemplar plates show a selection of naïve strains and transconjugants that were then measured.
**Figure S3:** Differences in the observed colony size of *Sp*3041 (pAGR96X) relative to the naïve strain *Sp3*041.
**Figure S4:** Growth curve of the *Sp*3041 naïve strain and its *Sp*3041 (pAGR96X) transconjugant in LB broth and M9 minimal media. (A) Growth in Luria‐Bertani broth. Shading denotes SD between replicates, whereas (B) shows growth in M9 (glucose) minimal salts. Measurements were taken over 24 h at 15‐min intervals.
**Figure S5:** Growth curve of the *Se*iDIA naïve strain and its plasmid transconjugants in LB broth and M9 minimal media. (A) Growth in Luria‐Bertani broth. Shading denotes SD between replicates, whereas (B) shows growth in M9 (glucose) minimal salts. Measurements were taken over 24 h at 15‐min intervals.
**Figure S6:** Growth curve of the *Se*477 naïve strain and its plasmid transconjugants in LB broth and M9 minimal media. (A) Growth in Luria‐Bertani broth. Shading denotes SD between replicates, whereas (B) shows growth in M9 (glucose) minimal salts. Measurements were taken over 24 h at 15‐min intervals. *Isolate where SD is not shown as replicate was contaminated.
**Figure S7:** Growth curve of the *Se*5.6 heat cured isolate and its plasmid transconjugants in LB broth and M9 minimal media. (A) Growth in Luria‐Bertani broth. Shading denotes SD between replicates, whereas (B) shows growth in M9 (glucose) minimal salts. Measurements were taken over 24 h at 15‐min intervals.
**Figure S8:** Predicted genomic islands and resistance genes on the chromosome of Se477. Colour indicates prediction methods used where blue; Island‐path DIMOB, orange SIGI‐HMM, green IslandPick and red the integrated result. Predictions were generated using IslandViewer 4. Boxed predicted islands signify erroneous (nil expression of genomic island genes) expression artifact identified in RNA transcriptome data (Figure 4). Full transcript and gene list with island annotations can be found in Supplementary Table S2.

## Data Availability

The data that support the findings of this study are openly available in BioProject: Serratia spp. comparative genomics at https://www.ncbi.nlm.nih.gov/sra/, reference number PRJNA1145474.
