# Supplementary material for: Conjugative Transfer of Disease‐Encoding Plasmid Variants in Serratia spp. Alter Production of Enzymes and Virulence Properties
Source: Environ Microbiol Rep. 2026 Feb 10;18(1):e70292. doi: 10.1111/1758-2229.70292 (PMC12890329; doi:10.1111/1758-2229.70292)
Supplement: Supplementary file 4 — Figure S1: Protease and DNase enzyme halo ratios and exemplar plates. Average ratio of protease/DNase expression with standard error for all isolates split by species (red recipient chromosome Sp; blue recipient chromosome Se). Exemplar plates show a selection of naïve strains and transconjugants that were then measured. Figure S2: Lipase and chitinase enzyme halo ratios and exemplar plates. Average ratio of lipase/chitinase expression with standard error for all isolates split by species (red recipient chromosome Sp; blue recipient chromosome Se). Exemplar plates show a selection of naïve strains and transconjugants that were then measured. Figure S3: Differences in the observed colony size of Sp3041 (pAGR96X) relative to the naïve strain Sp3041. Figure S4: Growth curve of the Sp3041 naïve strain and its Sp3041 (pAGR96X) transconjugant in LB broth and M9 minimal media. (A) Growth in Luria‐Bertani broth. Shading denotes SD between replicates, whereas (B) shows growth in M9 (glucose) minimal salts. Measurements were taken over 24 h at 15‐min intervals. Figure S5: Growth curve of the SeiDIA naïve strain and its plasmid transconjugants in LB broth and M9 minimal media. (A) Growth in Luria‐Bertani broth. Shading denotes SD between replicates, whereas (B) shows growth in M9 (glucose) minimal salts. Measurements were taken over 24 h at 15‐min intervals. Figure S6: Growth curve of the Se477 naïve strain and its plasmid transconjugants in LB broth and M9 minimal media. (A) Growth in Luria‐Bertani broth. Shading denotes SD between replicates, whereas (B) shows growth in M9 (glucose) minimal salts. Measurements were taken over 24 h at 15‐min intervals. *Isolate where SD is not shown as replicate was contaminated. Figure S7: Growth curve of the Se5.6 heat cured isolate and its plasmid transconjugants in LB broth and M9 minimal media. (A) Growth in Luria‐Bertani broth. Shading denotes SD between replicates, whereas (B) shows growth in M9 (glucose) minimal salts. Measurements we [file EMI4-18-e70292-s004.docx]

***
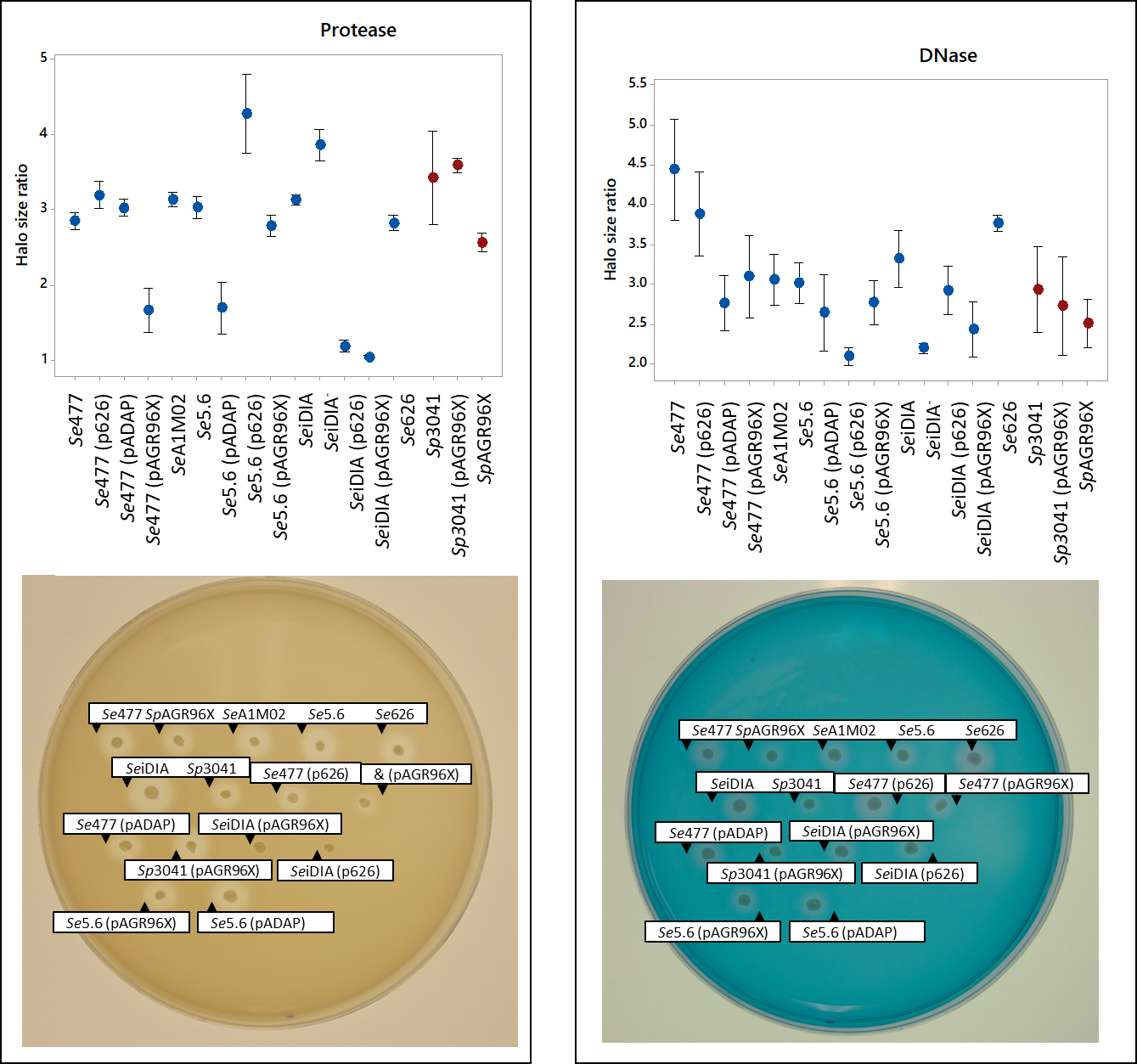
*Figure S1 Protease and DNase enzyme halo ratios and exemplar plates**Average ratio of protease/ DNase expression with standard error for all isolates split by species (red recipient chromosome *Sp*; blue recipient chromosome *Se*). Exemplar plates show a selection of naïve strains and transconjugants that were then measured.

***
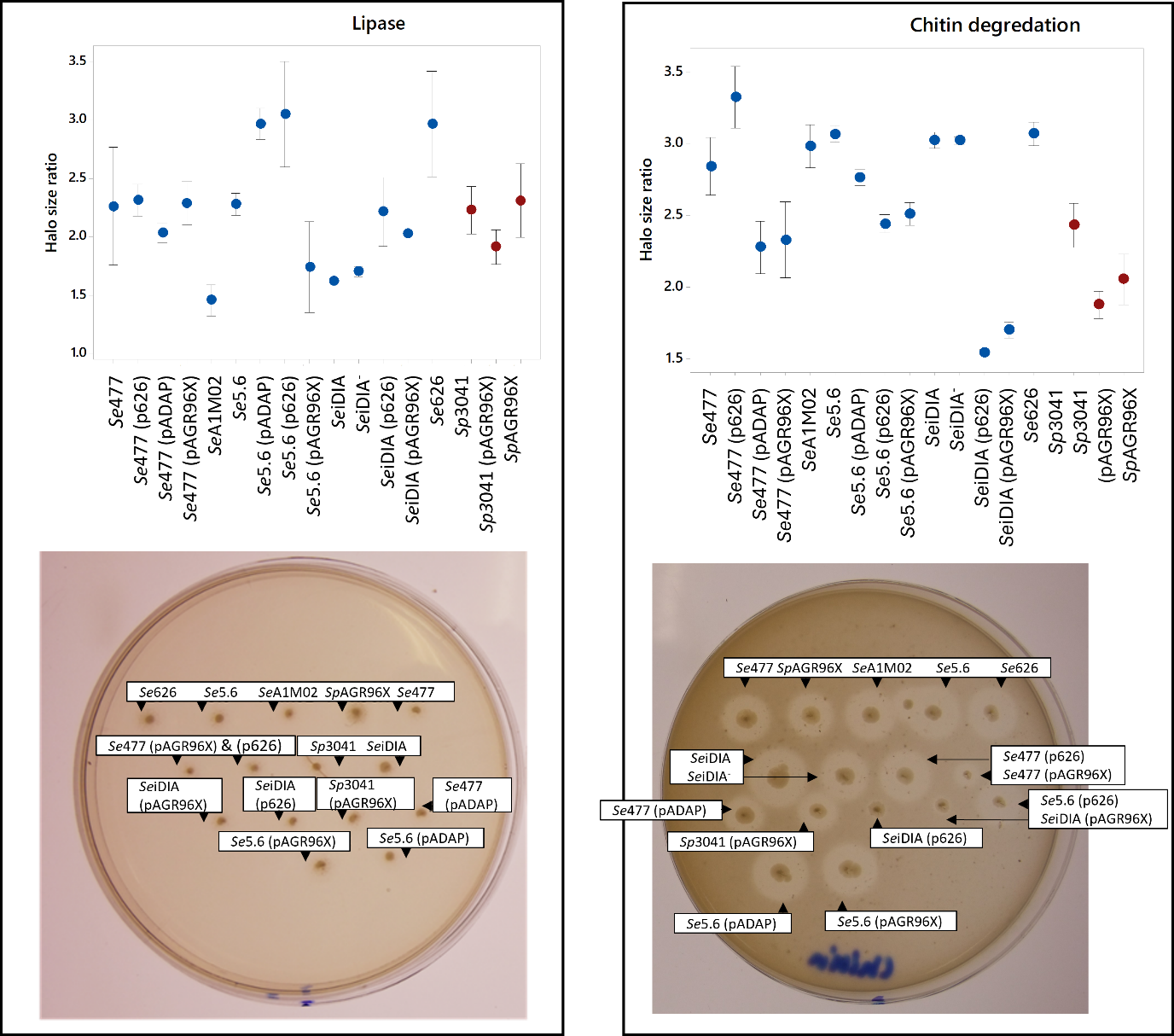
***

**Figure S2 Lipase and chitinase enzyme halo ratios and exemplar plates**Average ratio of lipase/ chitinase expression with standard error for all isolates split by species (red recipient chromosome *Sp*; blue recipient chromosome *Se*). Exemplar plates show a selection of naïve strains and transconjugants that were then measured.

***
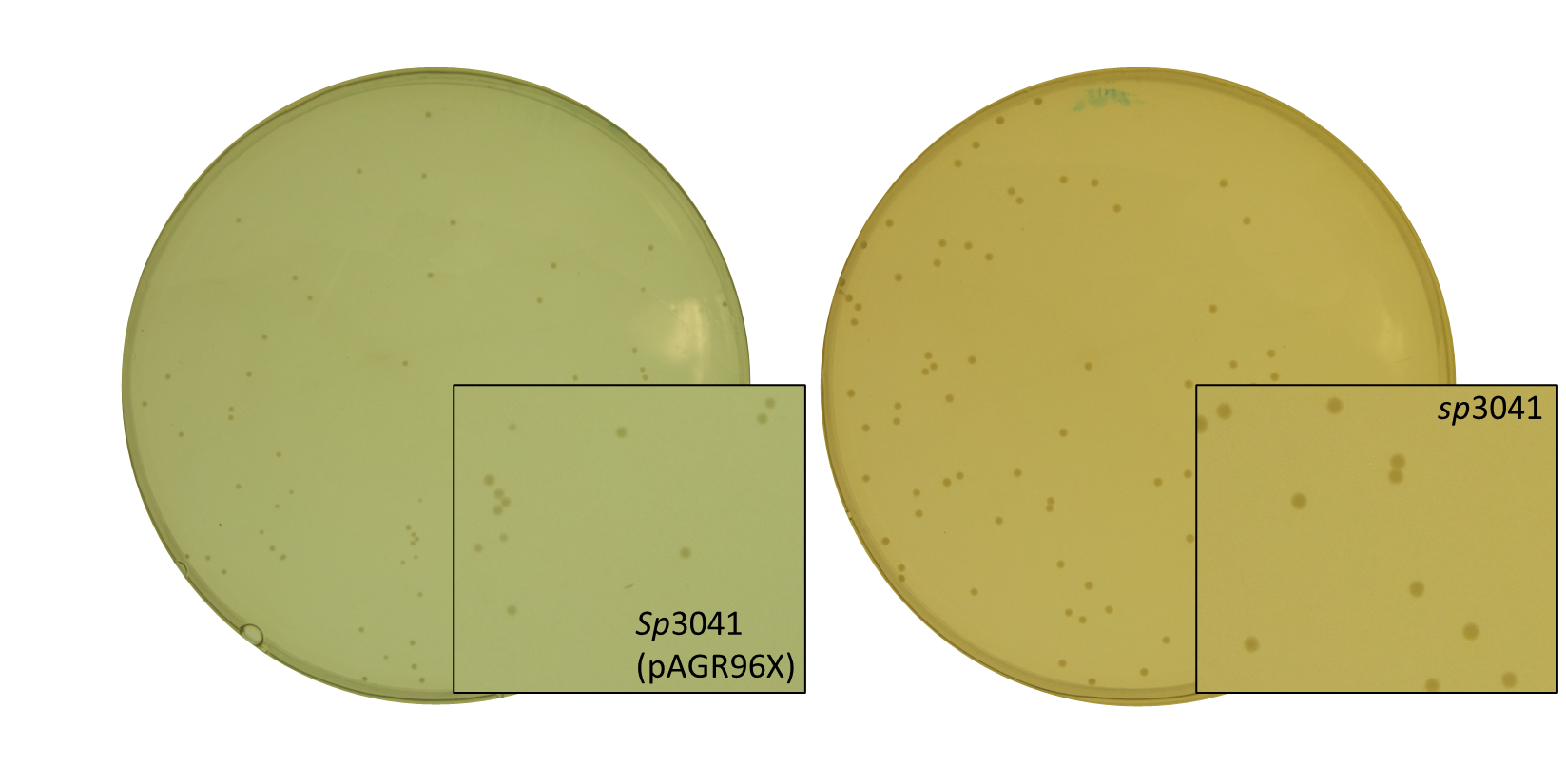
***

**Figure S3 Differences in the observed colony size of *Sp*3041 (pAGR96X) relative to the naïve strain *Sp3*041.**

**
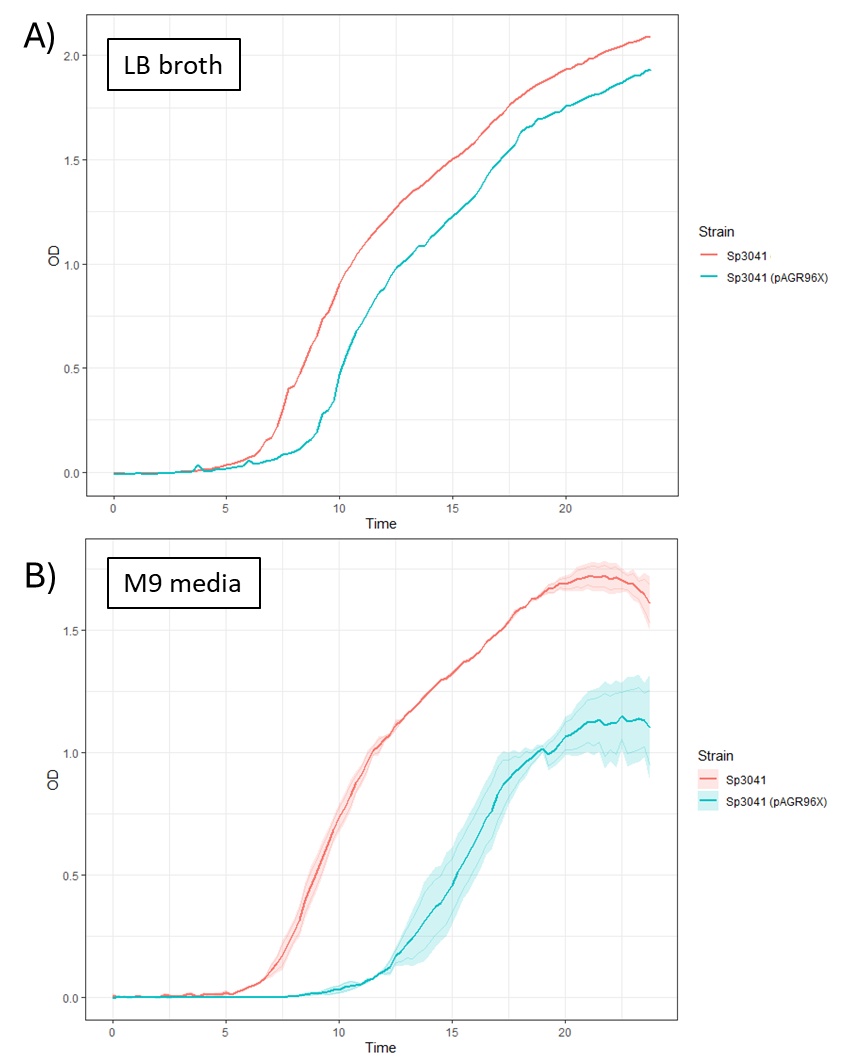
**

**Figure S4 Growth curve of the *Sp*3041 naïve strain and its *Sp*3041 (pAGR96X) transconjugant in LB broth and M9 minimal media.**A) Growth in Luria-Bertani broth. Shading denotes SD between replicates whereas B) shows growth in M9 (glucose) minimal salts. Measurements were taken over 24h at 15-minute intervals.

*
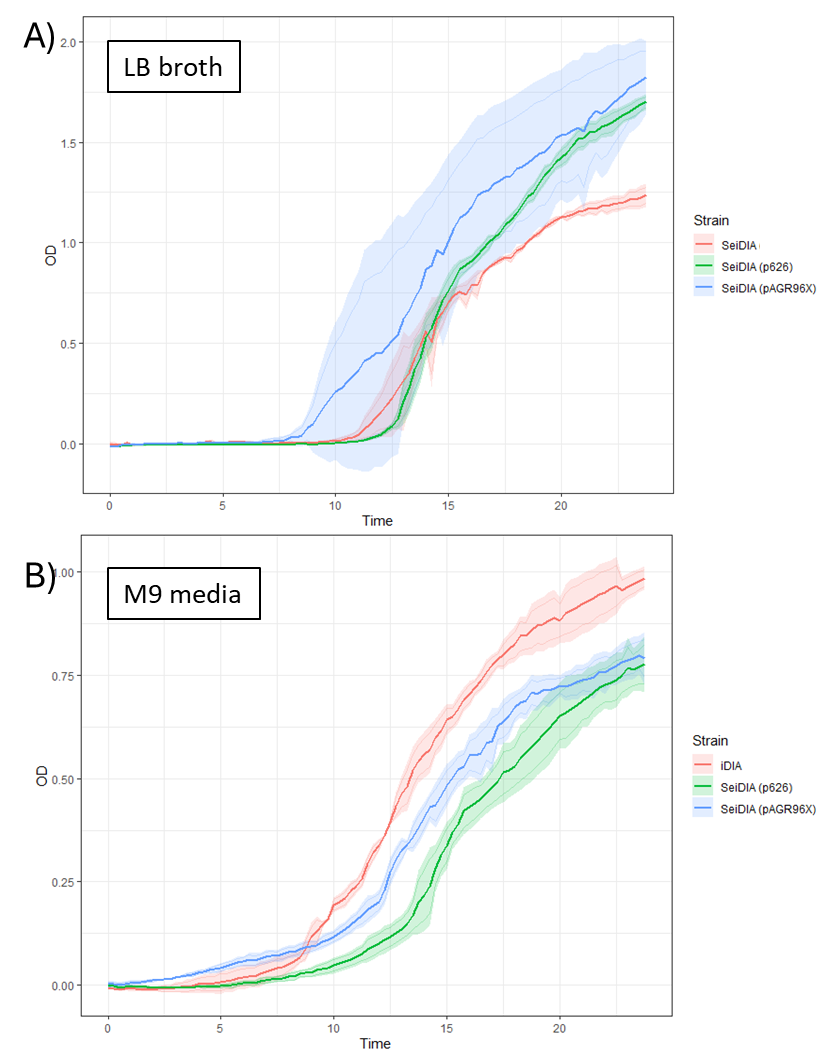
*

**Figure S5 Growth curve of the *Se*iDIA naïve strain and its transconjugants in LB broth and M9 minimal media.**A) Growth in Luria-Bertani broth. Shading denotes SD between replicates whereas B) shows growth in M9 (glucose) minimal salts. Measurements were taken over 24h at 15-minute intervals.

**
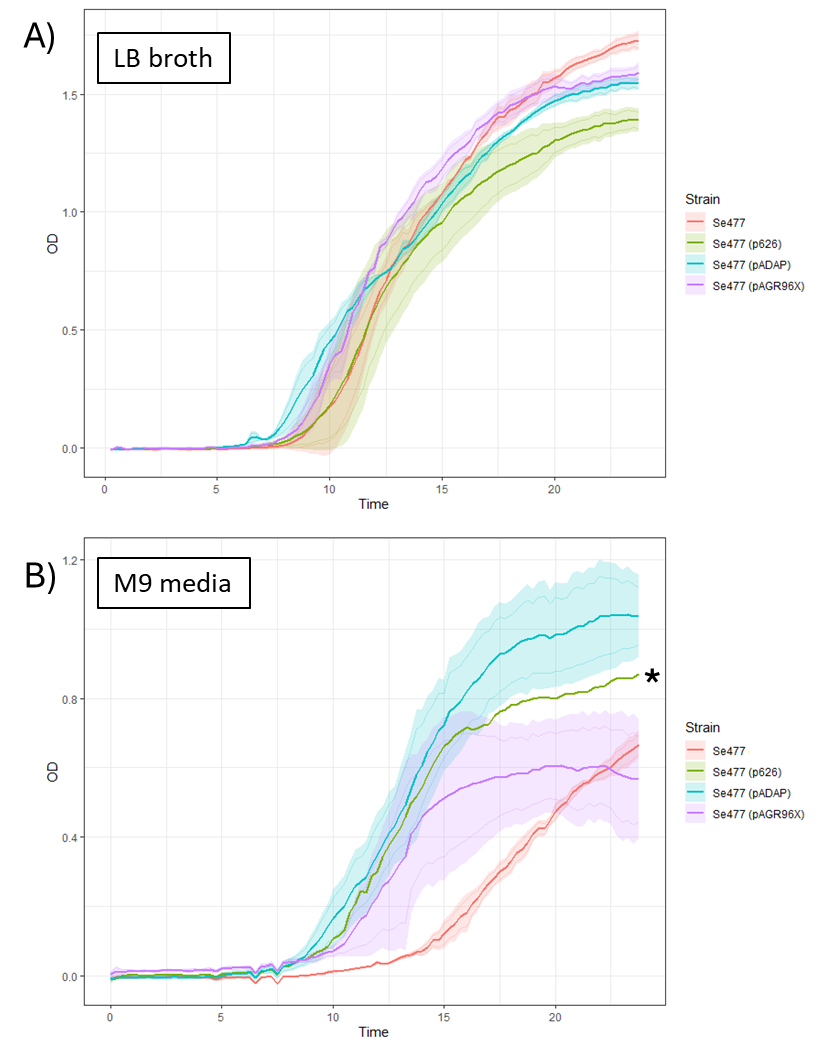
**

**Figure S6 Growth curve of the *Se*477 naïve strain and its transconjugants in LB broth and M9 minimal media.**A) Growth in Luria-Bertani broth. Shading denotes SD between replicates whereas B) shows growth in M9 (glucose) minimal salts. Measurements were taken over 24h at 15-minute intervals.
* denotes isolate where SD is not shown as replicate was contaminated.

*
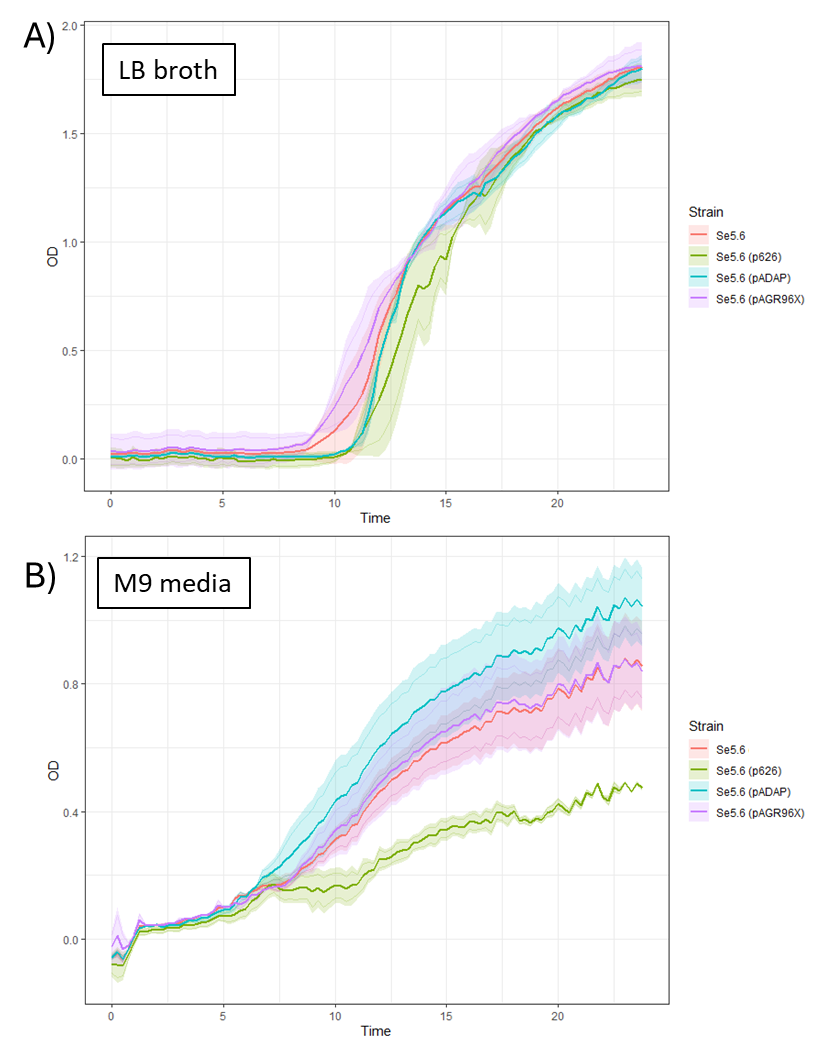
*

**Figure S7 Growth curve of the *Se*5.6 heat cured isolate and its plasmid transconjugants in LB broth and M9 minimal media.**A) Growth in Luria-Bertani broth. Shading denotes SD between replicates whereas B) shows growth in M9 (glucose) minimal salts. Measurements were taken over 24h at 15-minute intervals.

**
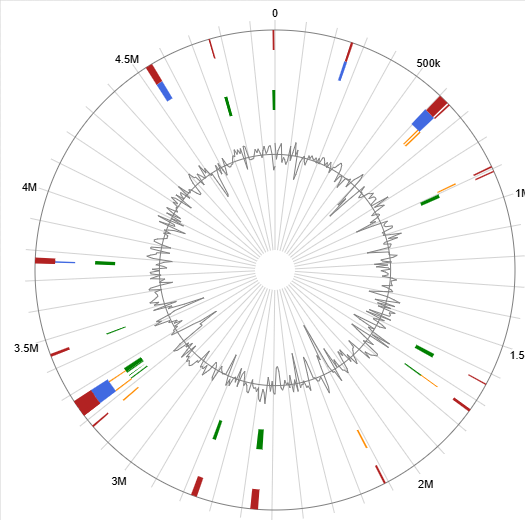
**

*S*

**Figure S8 Predicted genomic islands and resistance genes on the chromosome of *Se*477.** Colour indicates prediction methods used where blue; Island-path DIMOB, orange SIGI-HMM, green IslandPick and red the integrated result. Predictions were generated using IslandViewer 4. Boxed predicted islands signify erroneous (nil expression of genomic island genes) expression artifact identified in RNA transcriptome data (Figure 4). Full transcript and gene list with island annotations can be found in Supplementary Table S2.
